# The role of stromal cells in epithelial–mesenchymal plasticity and its therapeutic potential

**DOI:** 10.1007/s12672-024-00867-8

**Published:** 2024-01-20

**Authors:** Juanjing Wang, Junmei Peng, Yonglin Chen, M. I. Nasser, Hui Qin

**Affiliations:** 1 Hengyang Medical School, University of South China, Hengyang, 421001 Hunan China; 2 Guangdong Cardiovascular Institute, Guangdong Provincial People’s Hospital, Guangdong Academy of Medical Sciences, Guangzhou, 510100 Guangdong China; 3 School of Pharmaceutical Science, University of South China, Hengyang, 421001 Hunan People’s Republic of China; 4 The Hengyang Key Laboratory of Cellular Stress Biology, Institute of Cytology and Genetics, School of Basic Medical Sciences, University of South China, Hengyang, 421001 Hunan People’s Republic of China; 5 Key Laboratory of Ecological Environment and Critical Human Diseases Prevention of Hunan Province Department of Education, School of Basic Medical Sciences, University of South China, Hengyang, 421001 Hunan China

**Keywords:** EMT, Tumor metastasis, Tumor microenvironments, Stromal cells, Secretions

## Abstract

The epithelial–mesenchymal transition (EMT) is a critical tumor invasion and metastasis process. EMT enables tumor cells to migrate, detach from their original location, enter the circulation, circulate within it, and eventually exit from blood arteries to colonize in foreign sites, leading to the development of overt metastases, ultimately resulting in death. EMT is intimately tied to stromal cells around the tumor and is controlled by a range of cytokines secreted by stromal cells. This review summarizes recent research on stromal cell-mediated EMT in tumor invasion and metastasis. We also discuss the effects of various stromal cells on EMT induction and focus on the molecular mechanisms by which several significant stromal cells convert from foes to friends of cancer cells to fuel EMT processes via their secretions in the tumor microenvironment (TME). As a result, a better knowledge of the role of stromal cells in cancer cells’ EMT may pave the path to cancer eradication.

Tumor metastasis accounts for the majority of deaths of patients with cancer [1, 2]; however, the mechanisms still need to be elucidated before we can ultimately combat the cancer. Despite lethal cancer, which is The Sword of Damocles of most mammals, including human, is daunting in every aspect such as circadian rhythm [3, 4], microbiota [4], genetics [5–7], and metabolism [5, 8], encouragingly, there are commonalities in the cancer cell phenotype that are shared during tumor progressions such as acquired capabilities, emerging hallmarks, and enabling characteristics [9]. These commonalities, including but not limited to the emerging hallmarks of unlocking phenotypic plasticity [9–11], may open therapeutic windows for controlling tumor metastasis.

Cancer research has made pronounced advancements and expanded significantly over several decades, both preclinical and clinical. However, ‘The Sword of Damocles’ remains to take human lives [12]. Recently, an ever-growing number of studies have shown that cancer may be fought by targeting cell plasticity. Cell plasticity, also referred to as phenotype switching (for example, differentiation, de-differentiation, trans-differentiation and trans-determination), the opposite of cell identity, refers to a cell’s potential to adapt to a changing hostile milieu [11]. It is facilitated by a variety of molecules or molecular bases, including TP53 [13–15], Sox2 [15–17], androgen receptor (AR) [13–15, 17], and estrogen receptor α (ERα) [18, 19]. We will address trans-differentiation in this review, focusing on the effect of EMT on the plasticity and metastasis of tumor cells. Additionally, we will address the link between EMT and metastasis in various cancer-related environmental circumstances. By concentrating on EMT, whether directly on tumor cells or indirectly through the tumor microenvironment, such as stromal cells, new concepts and tactics for cancer therapy become accessible, therefore boosting established therapeutic processes.

## EMT is essential to physiology and pathology

EMT, sometimes called as epithelial–mesenchymal plasticity (EMP), is a developmental process that promotes the transition of epithelial cells to migratory mesenchymal cells and endows cells with stem-like potential [20, 21]. EMT is a vital driver of physiological processes such as embryonic development, tissue fibrosis, wound healing in normal tissues, and disease [22–25]. Specifically, EMT causes epithelial cells to separate from their connections to adjacent cells and alters their apical-basal polarity, revealing mesenchymal cell characteristics. Of interest is that both EMT and its reverse process, mesenchymal–epithelial transition (MET), occur throughout physiological and pathological processes such as embryogenesis, normal body development, wound healing, and organ fibrosis [26, 27].

In addition, EMT is also involved in the formation of tumors and is strongly linked with various features of cancer, including tumor development and metastasis [28–30]. More recently, a growing number of studies have uncovered that early tumor cells are definitely in epithelioid condition and progressively acquire more mesenchymal traits (that is, tumor-initiating capacity or stemness) as the tumor progresses [28, 30, 31]. Intriguingly, although EMT bestows upon cells the capacity of motility, its proliferative ability is decreased during tumor metastases, thus indicating that the MET program is also the other key player of tumor development and metastasis since MET-fueled metastatic outgrowth is essential for overt metastasis [31–33]. As EMT is controlled in many areas, cells that have undergone EMT in various tumor types or even at different stages of the same tumor play diverse metastasis roles despite being identical in shape, meaning that cell plasticity, specifically EMP, may be cancer cells’ ‘Achilles’ Heel’ and may provide strategies to against tumor metastasis [31, 34, 35], and this is why we pay more attention to playing. Therefore, a plethora of efforts have been made to elucidate the mechanism and to depict a much more comprehensive map of tumor cell EMT [36–38].

As mentioned above, tumor cells acquire interstitial characteristics through EMT, which endows the cells more motile and invasive, indicating that the malignant progression of all forms of cancer is linked to EMT [39, 40]. In recent years, EMT studies in tumor evolution have made substantial progress, providing compelling evidence of the unique relationship between EMT and metastasis. Given that a tumor mass is not composed solely of cancer cell but also includes surrounding cells, which are part of tumor microenvironments (TMEs), numerous regulatory elements within the TME influence this relationship. Therefore, the significance of EMT relying on the TME in playing a role in tumor metastasis is quite substantial.

## Drivers of EMT

**Fig. 1:**
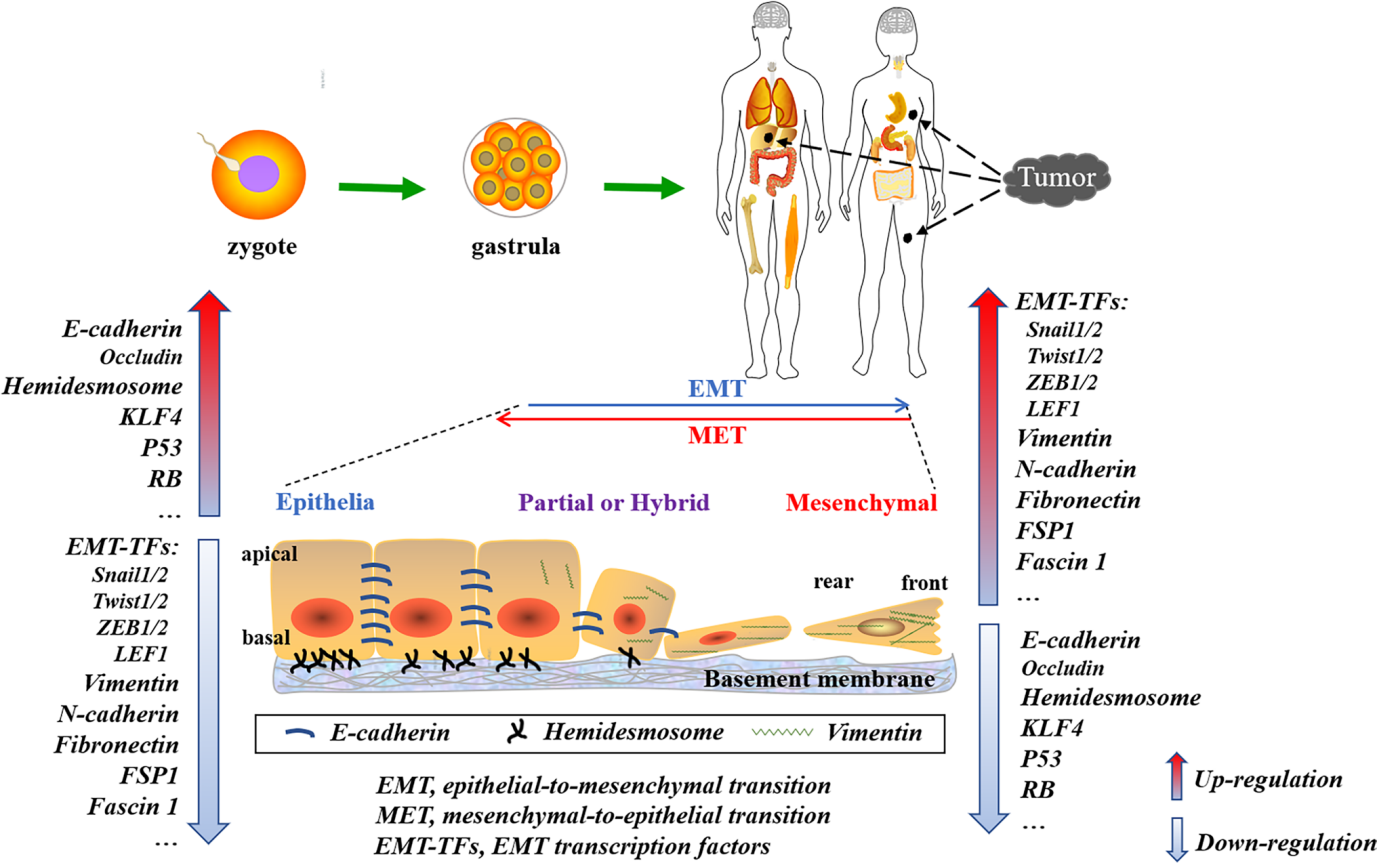
Overview of epithelial–mesenchymal plasticity in ontogeny and tumor progression. EMT is a highly dynamic and complex yet orchestrated programme in epithelial cells that lose some features such as down-regulation of the expression of P53, RB and E-cadherin, and gain some characteristics (for example, increased motility and stemness, along with enhanced expression of Vimentin, N-cadherin, FSP1 and Fascin1) to convert to mesenchymal phenotype. Vice versa, in the reverse process MET, cells increase their epithelia attributes and decrease mesenchymal ones. EMT-TFs tightly control these two processes to maintain development during ontogeny, whereas these normal programmes are also hijacked by cancer cells to evolve [24, 28]. Remarkably, cells with partial or hybrid EMT status possess both epithelial and mesenchymal attributions

During development, as a general biological process, EMT plays a key role, enabling cells of gastrula origin to migrate to foreign sites to form different tissues and organs of the body [25, 41]. Still, this normal process is also harnessed by cancer cells to evolve to survive in distinct settings (Fig. 1). Given that EMT governs both the body’s formation and the tumor’s progression, it is necessary to answer the question: How is the EMT activated?

To date, different EMT transcription factors (EMT-TFs) have been found. For example, Snail and Slug are zinc finger transcription factors, ZEB1 and ZEB2 are zinc finger E-box binding homeobox transcription factors, while Twist1 and Twist2 are basic helix–loop–helix transcription factors [42, 43] (Fig. 1). In addition to being a member of these three major EMT-TFs families, LEF1 was also implicated in EMT activation [44]. Thus, activating EMT-TFs is a critical method for controlling the expression of markers during tumor cell EMT. Based on this, we need to cautiously design and exert treatment regimens targeting EMT-TFs to minimize side-effects.

### EMT enhances the motility and resistance of tumor cells

The most distinguishing characteristic of cells that experienced EMT is increased motility, which facilitates tumor cell invasion and metastasis. Indeed, cancers often lose epithelial markers (for example, E-cadherin, p53, and RB) and exhibit mesenchymal markers such as Vimentin, N-cadherin and Fascin1 (see Fig. 1) before becoming invasive or circulating tumor cells (CTCs), hence facilitating tumor spread. EMT promotes tumor metastasis by increasing cell motility and by increasing tumor cells’ stem-like state, therapeutic resistance, and immune evasion [31, 45, 46].

EMT is also strongly associated with tumor cell drug resistance. Some drug-resistant tumor cells have an EMT phenotype, and those undergoing EMT are resistant to chemoradiotherapy in breast cancer and other malignancies [47, 48]. Twist1 confers resistance to chemoradiotherapy by inhibiting apoptosis in hypopharyngeal cancer cell lines [49]. Snail1 enhanced the capacity of head and neck squamous cell carcinoma cell lines to repair DNA damage caused by cisplatin by up-regulating the excision and repair of cross-complementary HIF-1α protein [50]. Additionally, Twist or Snail conferred resistance to gemcitabine on pancreatic cancer cells by modulating the expression of drug-inactivated enzymes and/or transporters [51]. Due to the fact that EMT occurs in a variety of different cancer types through various pathways, cancer cells might develop resistance to chemoradiotherapy or drugs, and inhibiting EMT can improve treatment sensitivity. Taken together, these observations strongly imply EMT plays a paramount role in tumor progression and is, to some extent, responsible for the vast majority of drugs failures in the clinic.

### EMT and tumor stem cells

Cancer stem cells (CSCs), also known as tumor-initiating cells (TICs) or CTC, are tiny subpopulations of tumor cells that play a pivotal role in carcinogenesis and progression, as these subpopulations that are undergoing EMT possess stem cell-like characteristics, namely be of the ability to self-renew and resistance to a range of therapeutic agents [31, 38, 52–56]. It is noteworthy that the signaling pathways engaged during EMT are strikingly comparable to those that drive CSCs, including Wnt/β-catenin, TGF-β/Smads, and other signaling pathways that are required for CSC self-renewal and maintenance [38]. Accordingly, these findings explain why a minute proportion of CSCs are produced during EMT.

Epithelial cells are normally polarized apical-basal axis and are connected by tight junctions, adherens junctions, desmosomes, and hemidesmosomes. E-cadherin, a core molecule on the cell surface, is responsible for adhesion and controlling and/or maintaining cells’ epithelial identity or phenotype [38, 57]. This link is critical for epithelial tissue’s structural integrity and solid tumor’s formation. Following EMT activation, the characteristic polygonal and pebble-like shape of epithelial cells or cobble-stone cell islands phenotype is lost, resulting in cells being more readily separated from epithelial tissues. A recent study corroborated that the cellular adhesion and polarity of colorectal carcinoma were diminished when EMT occurs. Accordingly, a drop in the expression of epithelial markers was observed [58]. On the other side, interstitial features are acquired when cells develop a fusiform mesenchymal shape, which is accompanied by a rise in the mesenchymal markers N-cadherin, Fascin1, Fibroblast specific protein 1 (Fsp1) and Vimentin [42, 59–63] (Fig. 1).

Mesenchymal cells exhibit mobility and migration due to interstitial markers and changes in cell shape. Due to the gain of front-rear polarization nature instead of apical-basal polarization and the absence of intercellular connections, individual mesenchymal cells may migrate across the extracellular matrix (ECM). Reduced E-cadherin expression and increased N-cadherin expression decrease cells’ adhesion ability and increase cell mobility, allowing cells to migrate and invade, which are indicative of EMT [64, 65]. It is not difficult to observe that EMT fundamentally transforms cell phenotypes by altering the expression of epithelial and mesenchymal phenotypic markers.

**Fig. 2:**
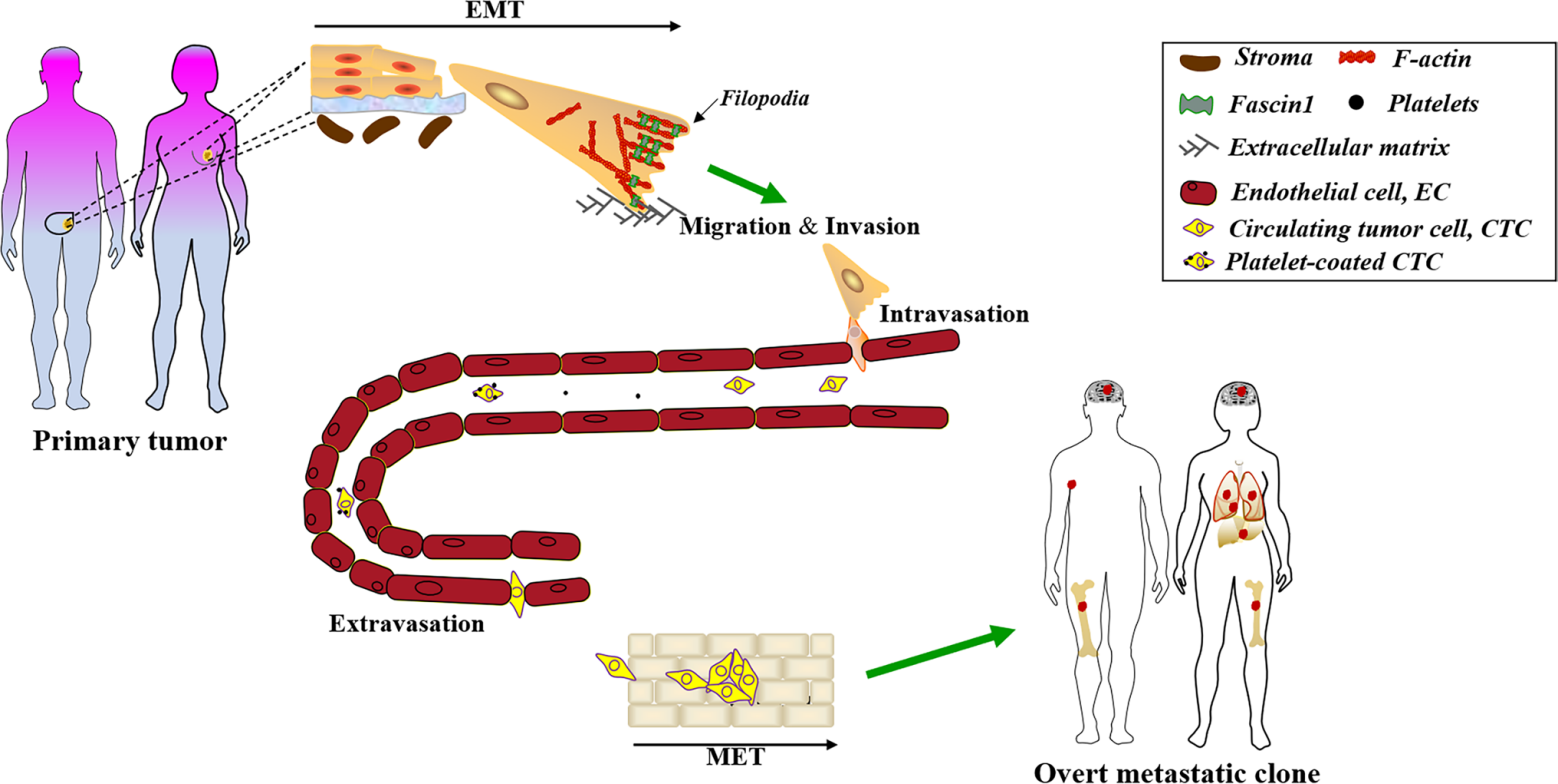
The paradigm of EMT promotes tumor cells migration, invasion and metastasis. Multiple rounds of EMT process confers cancer cells with elevated expression of Fascin 1 to form filopodia, which mediates the cancer cells departing from primary lesion to entry into circulation both in the ‘*Linear progression model*’ and in the ‘*Parallel progression model*’ [70]. Then, CTCs were coated by platelets to diminish blood flow shear force avoid killing by immune cells. The cells conveyed by circulation subsequently extravasate to the parenchyma of foreign organs, followed by a MET to undergo metastatic outgrowth (the overt metastatic clone)

On the other hand, mesenchymal cells may return to an epithelial condition through the reversal of EMT process, namely the MET. These changes are intimately connected with tumor differentiation [66]. By reducing E-cadherin expression, initial cancer cells lose their cellular adhesion and gain mesenchymal features, converting them into migratory and invasive cancer cells. These cancer cells will pierce the basal membrane, enter the blood through blood arteries, and then overflow into the area of metastasis, creating micrometastasis on the target organ’s parenchyma [67–69]. Once reaching the metastatic location, tumor cells undergo MET to revert to their epithelial origin, forming secondary malignant tumors. The back-and-forth conversion between EMT and MET will eventually kill the cancer patients who are bearing a tumor mass of approximately 1 kg or harboring 10^12^ to 10^13^ cancer cells [70]. This diagram depicts the whole process of tumor cell migration through EMT (Fig. 2).

### EMT mediates immune escape of tumor cells

When cancer cells and tumor stem cell-like cells undergo EMT, they display immunosuppressive and immune-resistant properties. Cancer cells may interact with various immune cells and other stromal cells in tumor tissue, resulting in the formation of an immunosuppressive microenvironment. Indeed, snail-induced EMT may promote melanoma progression by altering T cell-mediated immune suppression. Additionally, EMT-producing cells may impede the killing function of cytotoxic T cells by disrupting immunological synapses. EMT may potentially help in immune evasion by boosting the expression of immunological checkpoint proteins on tumor cells, such as programmed-cell death ligand 1/2 (PD-L1/2) and B7-H3 [71–74].

## Stromal cells and their function in tumor EMT

Before understanding the role of stromal cells in tumor EMT, it is necessary to review the so-called ‘seed and soil’ theory of tumor metastasis, which was proposed by Steven Paget in 1889 when he analyzed the corpses of 735 breast cancer patients and combined his observation with evidence obtained from other cancers [75]. The theory underscored the contribution of the ‘seed,’ namely the disseminated tumor cells (DTCs) or CTCs, and the ‘soil,’ a favorable environment of foreign tissue or organs for cancer cells to promote tumor metastasis. This conception argued that the ultimate metastatic lesion formation is established upon the bi-directional selection between the seed and the soil, meaning that the DTCs and foreign environment are compatible. Although several ideas have questioned the ‘seed’ and ‘soil’ notion [76], the idea provides a plausible explanation for metastasis organotropism [69, 77, 78]. Additionally, the hypothesis also leads to the eruption of research on the TMEs, which include, but are not limited to, stromal cells (for example, cancer-associated fibroblasts, infiltrating immune cells and endothelial cells), as well as non-cellular components such as matrix proteins, nutrients and the concentration of oxygen [79–82].

Recently, studies on organ transplantation demonstrated that the distinct outcome of both cancer-related death and metastasis-free survival was observed in donors and organ transplant recipients, particularly in recipients who received the same donor’s organ [83–85]. This compelling evidence further corroborates Stephen Paget’s notion suggests that the TME, often referred to as the ‘soil,’ plays both anti- and fuel-cancer function. This mutually opposing dual role depends on the capacity of seed adaption to the unfriendly TMEs and the tumor stage. For example, stromal cells may function as barriers and accessories in and early and advanced stage of cancer, respectively. Fortunately, an emerging conception of ‘Dependence Receptors’, such as Deleted in colorectal carcinoma (DCC) [86], Notch [87], and c-Kit [88], may provide a further explanation for the dual role of TMEs in tumor development [89]. Considering both the ‘seed’ and ‘soil’ notion and the ‘Dependence Receptors’ theory, it is easy to imagine the dual function of stromal cells may rely on whether ligands bind to their receptors. Building on this, we will mainly focus on the effects of secretions originating from stromal cells on EMT activation in tumor cells.

## Promotion of tumor EMT by cancer-associated fibroblasts

As one of the three major stromal cells, cancer-associated fibroblasts (CAFs) are an integral part of the TME and play a critical role in remodeling the extracellular matrix, malignant transformation, and tumor metastasis [31, 90, 91]. CAFs are produced from various cell types, including mesenchymal fibroblasts, epithelial–mesenchymal transformation cells, vascular endothelial cells, differentiated mesenchymal cells from the bone marrow, adipocytes, and stellate cells [91, 92]. Mesenchymal cells and fibroblasts are the primary origin of CAFs. These CAFs from diverse sources are regularly recruited to the tumor’s periphery and act as a regulator throughout tumor growth. CAFs exert several regulatory functions on tumor evolution. To begin, CAFs can release tumor growth factors, encouraging tumor cell proliferation. Second, CAFs can drive endothelial progenitor cells into tumor tissues, hence promoting the creation of intratumor blood vessels. Additionally, CAFs have been shown to play a critical role in tumor development and metastasis in vivo. Multiple studies have shown that EMT regulation is the most critical among the numerous ways by which CAFs regulate tumor growth [93, 94]. The synergistic effect of a variety of cytokines and growth factors produced by CAFs is a critical mechanism by which CAFs induce EMT in cancer cells. These factors include transforming growth factor β (TGF-β), interleukin 6 (IL-6), vascular endothelial growth factor (VEGF), Wnt, and hepatocyte growth factor (HGF), all of which are generated in a paracrine manner by CAFs [38, 94–96]. Collectively, these results indicate that CAFs are known to induce EMT in cancer cells through a paracrine mechanism and imply targeting CAFs may be a better choice for cancer treatment.

### Transforming growth factor

Among the cytokines generated by CAFs, the most extensively researched is TGF-β, which plays a critical role in developing many types of malignancies [97, 98]. Mesenchymal fibroblasts may release TGF-β and stromal cell-derived factor-1 (SDF1), which function in concert through distinct signaling pathways to sustain mesenchymal fibroblast differentiation into myofibroblasts during cancer development. TGF-β levels were much greater in myofibroblasts than in normal stromal fibroblasts [99].

**Fig. 3:**
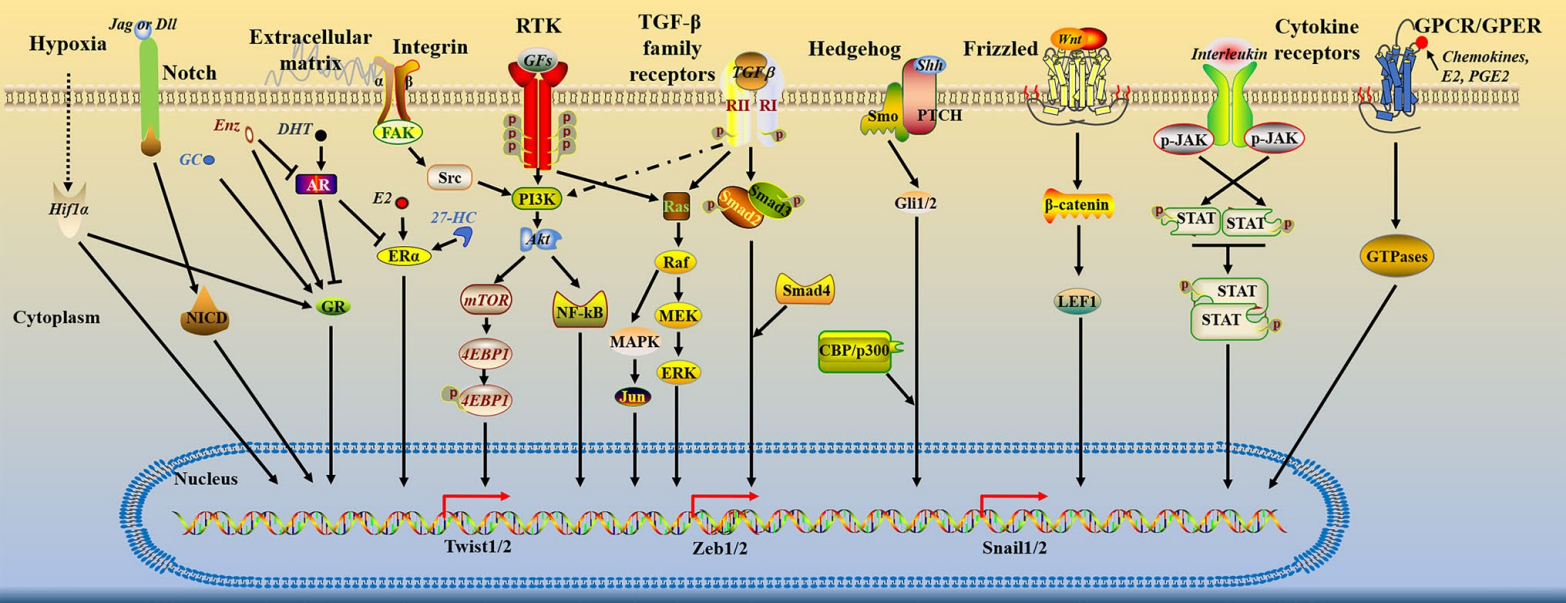
The master pathways implicated in EMT activation. Several signaling pathways regulate EMT-TF induction, which is most evident in the cancer setting. In this scenario, cEMT or pEMT is primarily responsible for this process. By activating of the Jagged/Delta-Notch-NICD axis and the Wnt-Frizzled-β-catenin-LIF1 pathway, the ligands Jagged/Delta and Wnt induce EMT processes. Regarding the signaling pathways involved in the convergence of events that facilitate the EMT process, it has been observed that HIF-1α can recruit the glucocorticoid receptor (GR). The GR is a hormone receptor activated by glucocorticoids (GC) and certain androgen deprivation agents like enzalutamide (Enz). However, the activity of GR is inhibited by the AR. This recruitment of GR by HIF-1α enhances the expression levels of EMT-TFs in cells once they detect a hypoxic environment. The involvement of cytokine receptors, Sonic-Hedgehog, and GPCR/ GPER signaling pathways in the process of EMT has also been suggested (The image was adapted from Ref. [38])

TGF-β, which CAFs release, influences EMT by modulating gene transcription in the nucleus through many distinct signaling pathways. The TGF-β/Smads signaling pathway is critical for regulating EMT-related genes in cancer cells among several signaling pathways. TGF-β is recognized by two distinct receptor types: type I and type II receptors (TRI and TRII, respectively). TGF-β dimers bind to its receptors (TRI and TRII) on the cell surface, inducing TRII phosphorylation followed by phosphorylating TRI through its serine and threonine kinases domain. Subsequently, the receptor-phosphorylated Smad2/3 (R-Smads) is recruited and phosphorylated by active type I receptors, and these receptor-activated Smads (R-Smads) form protein complexes with the ubiquitous Smad4 (Co-Smad). The activated Smads complex is transported to the nucleus and cooperates with other transcription factors to co-regulate the transcription of target genes [100–102] (Fig. 3). Additionally, TGF-β may exert its effect via regulating MAPK, PI3K/Akt, and other cancer-related signaling pathways through non-classical signaling pathways [103]. Similarly, TGF-β is plentiful in CAFs conditioned media and may promote EMT in bladder cancer cells by activating Smad2, which activates classical TGF-β signaling [104, 105]. TGF-induced EMT resulted in the overexpression of genes associated with Snail1, ZEB1, and ZEB2. It also leads to the down-regulate of the epithelial marker E-cadherin and up-regulation of the mesenchymal markers such as vimentin and N-cadherin, ultimately EMT transformation [105].

### Interleukin-6

IL-6, an inflammatory factor, is another key cytokine released by CAFs that promotes EMT in tumor cells. CAFs induce EMT by secreting IL-6 and promoting cancer cell migration [96]. Signals travel from outside the cells to the nucleus following JAK2/STAT3 signaling pathway, which is activated when IL-6 binds to the receptor on the cell membrane. Specifically, the IL-6 signal by activating JAK2 protein kinase mediates the phosphorylation of STAT3, which is then translocated from the cytoplasm into the nucleus to regulate the expression of EMT-related and other genes [96, 106]. Similarly, IL-6 may alter the phenotype of human peritoneal mesothelial cells (HPMCs) in vitro from pebble-like to fibroblast-like, which was an EMT feature [107]. Also, IL-6 produced from CAFs may develop resistance to cisplatin in non-small cell lung cancer (NSCLC) through promoting EMT [108]. According to a study, CAFs boost TGF-β mediated EMT in ovarian cancer by increasing IL-6 production through the JAK2/STAT3 pathway, which suppresses apoptosis and results in higher resistance to paclitaxel [109]. It is clear from these findings that the mechanism by which CAFs secrete IL-6 to induce EMT in tumor development might enhance tumor incidence and spread as well as tumor medication resistance. More CAF-secreted IL-6 is expected to enhance cancer cell migration and EMT-specific activities shortly.

### Other secretions

Beyond the factors that were mentioned above, other growth factors and chemokines secreted by CAFs can also induce EMT: in pancreatic and breast cancer, VEGF produced by CAFs causes EMT by increasing the expression of EMT-related genes such as Snail and Twist [110, 111]; matrix metalloproteinases (MMPs) such as MMP2, MMP3 and MMP9 generated by CAFs degrade E-cadherin through the Rac1b/ROS pathway to fuel β-catenin entry into the nucleus to induce EMT and improve HCC cell motility [111, 112]; gastric cancer cell metastasis was also increased through the C-MET (HGF receptor) pathway activation when hepatocyte growth factor (HGF), secreted by CAFs, was binding to C-MET [113]. Indeed, the HGF ligand triggers EMT in tumor cells by activating many intracellular signaling pathways [114].

In addition to these mentioned growth factors and chemokines, other secretions also generated by CAF, including Sonic Hedgehog (Shh) [115], Netrin-1 [116], Jag/Dll [117], Wnt [118], 17 β-estradiol (E2) [119] and α-smooth muscle actin (α-SMA) [120], can also favour EMT activation in tumor cells.

## Tumor-infiltrating immune cells and EMT

Immunotherapy, a powerful and safe targeting therapeutic strategy, was elicited by the ground-break discoveries of the programmed-cell death protein 1 (PD1) and cytotoxic T lymphocyte-associated protein 4 (CTLA4) by Tasuku Honjo and James P. Allison, respectively [121, 122]. Since then, a revolution in cancer treatment has targeted the tumor immune microenvironment (TIME). TMIE is part of the TMEs and mainly consists of infiltrating immune cells, including tumor-associated macrophages (TAMs), marrow-derived suppressor cells (MDSCs), and T lymphocytes [123]. Beyond these three types of cells, natural killer (NK) cells, neutrophils, and dendritic cells are also members of the tumor-infiltrating immune cells [124–128]. Accordingly, a better understanding of the effects of tumor-infiltrating immune cells (TIICs) in TMEs on tumor progression will benefit for deciphering the complexity of cancer and developing novel therapeutic schemes. In this section, we will mainly focus on the influence of TAMs, MDSCs, and T-cells on tumor EMT programme.

### Induction of tumor EMT by tumor-associated macrophages

**Fig. 4:**
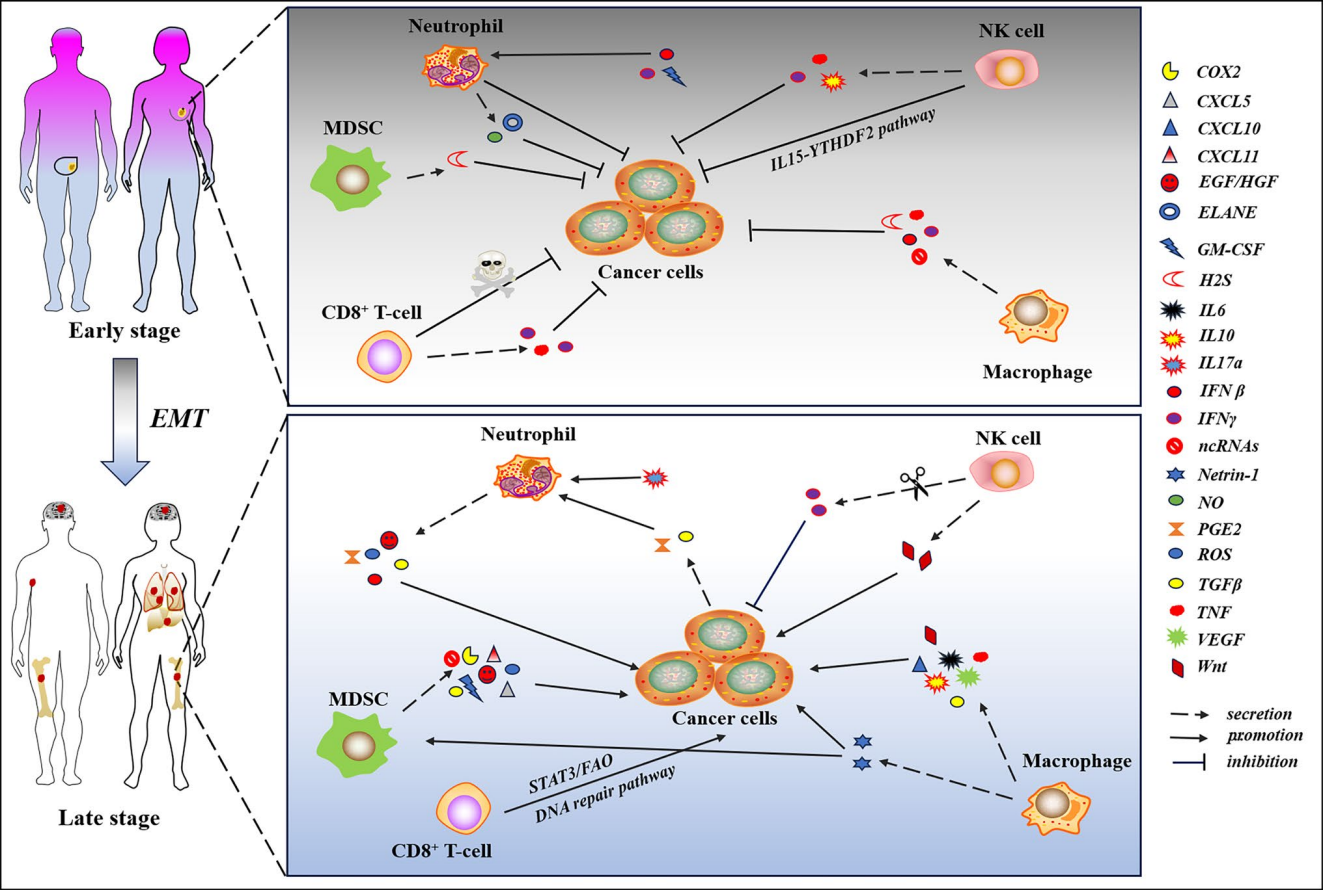
The dual roles of immune cells within TME. The schematic illustration represents communication networks between immune and cancer cells that serve as foes or accomplices in early- and late-stage. Briefly, immune cells in TME secrete a range of factors (for example, IFNβ, IFN*γ*, and TNF) and unleash gasotransmitters such as H2S and NO or employ pathways to kill cancer cell in the early stage of the disease. Unfortunately, these foes of cancer cells, following rounds of EMT, stepwise became friends with cancer cell to conspire in fuelling tumor progression by secretions or pathways. For instance, TGFβ, a factor of the suppressive microenvironment generated both by neutrophils and cancer cells, exacerbates the suppressive microenvironment that promotes cancer cell immune evasion, survival, and expansion. In addition, NK cells in TME can plunge the production of the IFN*γ*, which was utilized to inhibit cancer in the early-stage, to block the inhibitory effect in the late-stage. Meanwhile, NK cells also inreased the secretion of Wnt to fuel cancer cell growth in contrast to the direct cytotoxic effect of CD8^+^ T-cell in the early-stage, which was highjacked by cancer cells to facilitate cancer cell escape from adverse milieu through stat3/FAO and DNA repair pathways to survival

Induction by TAMs is another dramatic mechanism for EMT. As a highly malleable immune cell, macrophages are critical for immunological metabolism, chronic inflammation, and tissue homeostasis [129, 130]. TAMs are macrophages that move to tumor stroma and are often the most numerous immune cells highjacked by cancer cells to enter into the TMEs to distort their properties from anti- to pro-cancer [131] (Fig. 4). They play a role in the genesis of cancer, the generation of drug resistance, and immunological escape in a variety of malignant tumors, and are linked with a poor prognosis [130, 132]. With respect to the pro-cancer role of TAM in TMEs, a report shows TAMs accelerate cancer progression by increasing stromal remodeling, suppressing adaptive immunity, and boosting angiogenesis and EMT [133].

TAMs have distinct behaviors in various malignancies, often manifesting as the polarization of anti-inflammatory and pro-tumor phenotypes, including the M1 and M2 subtypes [134–136]. M1-type macrophages have been shown to slow down tumor growth, but M2 macrophages release a range of cytokines that induce EMT and immunosuppression, TGF-β, IL-1β/6/10, CXCL10, HIF-1, and VEGF are a few of these cytokines [137–140]. These cytokines act as a suppressor of adaptive immunity during the onset of EMT. M2 macrophages and regulatory T cells (T-regs) are more abundant in tumor stroma formed from mesenchymal cell lines than epithelial cell line-derived cancers [71]. Under hypoxia, Hif-1α stimulates the release of CCL-20 by mesenchymal cancer cells, which in turn increases the expression of indoleamine 2,3-dioxygenase (IDO) in TAMs, ultimately impairing the function of CD4^+^ and CD8^+^ T cells in hepatocellular carcinoma [141]. Likewise, inhibition of mTORC1/mTORC2 may suppress EMT and down-regulate TAM recruitment and PD-L1 expression, therefore boosting immunity against lung cancer [142]. The M2 subtype of macrophages was also encouraged to polarize in gastric cancer-derived mesenchymal stromal cells (GC-MSC). The GC-MSC-induced M2-type macrophages then increased the expression of interstitial marker genes vimentin, fibronectin, and Slug. Conversely, E-cadherin expression was dramatically reduced. Hence, GC-MSC-induced M2 macrophages promote gastric cancer cell EMT and thus tumor spreading. Ontogeny-induced M2-type macrophages play a critical role in TAM formation and immunosuppression [143]. Thus, inhibiting anti-tumor M1-type macrophages skew to pro-tumor M2-like macrophages in TME may suppress tumor progression by aiming to EMT and provide a novel target for cancer treatment.

### TAMs induce other cytokine pathways of EMT

Several different mechanisms exist for TAMs to produce cytokines to drive tumor EMT. In a paracrine way, similar to CAFs, TAMs release Wnt2b in hepatocellular and thyroid carcinoma cells, inducing EMT [144, 145]**.** TAM may also release tumor necrosis factor (TNF), and the synergistic effect of TNF and TGF-β has been shown to enhance EMT in colon cancer [146].

Mesenchymal-like breast cancer cells attract TAMs by the secretion of granulocyte–macrophage colony-stimulating factor (GM-CSF), while TAMs recruited by surrounding tumor cells produce CCL18. Therefore, metastasis is facilitated by inducing EMT, and patient survival is, no doubt, shortened [147]. Additionally, IL-6 produced by TAMs operates on the COX-2/PGE2 signaling pathway and promotes EMT by stimulating the transfer of β-catenin from the cytoplasm to the nucleus to regulate the EMT-related gene expression [148].

### Suppressor cells of bone marrow origin and EMT activation

Bone marrow-derived suppressor cells (MDSCs) are a member of TIICs and cause EMT activation. Although MDSCs are suppressor cells, De Cicco et al. found that MDSCs play an anti-cancer role through the excretion of H_2_S, a gasotransmitter, in the early-stage [149], similar to TAM (see Fig. 4). MDSCs are immature bone marrow cell types that have been shown to induce EMT and accelerate non-small cell lung cancer metastasis via the CCL11-ERK/AKT-EMT axis [150]. A recent work showed that MDSCs are attracted into primary tumors by myeloid-specific chemical attractant SPARC, an extracellular matrix (ECM) component, thereby inducing the EMT programmes in high-grade breast cancer cells [151]. Once recruited, MDSCs cluster around the original tumor, promoting EMT, creating cancer stem cell-like cells, and disseminating cancer cells through the activation of TGF-β, COX2, and the secretion of EGF and HGF [152].

MDSCs, as variable myeloid cell morphologies, may contribute to immunosuppression in TME through EMT. MDSCs suppress a variety of T cells, nNK cells, and dendritic cell activities [153]. MDSCs have osmotic and inhibitory properties regulated by the inducible enzymes CXCL2 and COX-2 in mammary and colorectal cancer [151, 154], respectively. Snail increased CXCL2 expression through NF-kB signaling and encouraged MDSC recruitment into the tumor microenvironment, suppressing CD8^+^ T cells and encouraging ovarian tumor development [155]. Similarly, upregulation of β-catenin/TCF4 and COX-2 in nasopharyngeal cancer increases MDSC-mediated EMT. Additionally, COX-2 has been shown to regulate the interaction of cancer cells with MDSCs, hence promoting metastasis [156]. As such, MDSC-induced EMT contributes to tumor cell migration and this is comparable to how CAFs-induced EMT plays a role in tumor evolution.

### T lymphocyte

Immune cells also contribute to EMT and tumor metastasis. Numerous immune cells accumulating in the tumor stroma interact with neighboring cancer cells, reactivating latent EMT processes. Although the general view is that the cytotoxic CD8^+^ T cells are of anti-tumor capabilities and serve as the mainstay of immunotherapy, this subset of cells can also induce EMT and increase tumor metastasis as representative immune cells in immune surveillance and immune education. For example, the melanoma-associated antigen C3-facilitated EMT and metastasis in esophageal squamous cell carcinoma are mediated by PD1^+^ CD8^+^ T cells infiltrating the tumor [157]. In addition, CD8^+^ T cells were discovered to promote EMT in vivo, resulting in the formation of mesenchymal breast cancer cells with stem-like characteristics [158] (Fig. 4).

CD4^+^ T cells, in addition to CD8^+^ T cells, may initiate the EMT process. Importantly, reduced interferon-gamma expression was seen in PanINs that were densely surrounded by CD4^+^ T cells, in contrast to PanINs that were not tightly surrounded by CD4^+^ T cells [159]. Furthermore, co-culture with activated effector CD4^+^ T cells in vitro results in the down-regulation of E-cadherin in pancreatic ductal epithelial cells. The epithelial cells acquire a fusiform mesenchymal appearance with vimentin and ZEB1 expression [160]. Additionally, researchers discovered that CD4^+^ T cells, the primary source of IL-6, triggered the EMT process in clear-cell renal cancer [161]. Although the mechanism by which T cells induce EMT remains unknown, cytokines generated by activated effector T cells (e.g., IL-6, TNF, and TGF-β) are known to promote EMT.

### Other immune cells

As forementioned, NK cells, neutrophils, and dendritic cells are part of TIICs, which, together with suppressive factor of TME such as TGF-β constitute the TIME that plays a predominant role in tumorigenesis and progression. An accumulating body of evidence disclosed that TIICs play both pro- and anti-cancer roles during tumor development, akin to their partner stromal cells. The pro- or anti-cancer activity of TIICs also depends on the stage of tumor, as shown in Fig. 4. Considering the auxiliary role of dendritic cells in immune activities, we will, therefore, briefly discuss the functions of NK cells and neutrophils in tumor progression here.

As an important player in the TME, NK cell undoubtedly plays a key role in tumor evolution (Fig. 4). Yu’s group demonstrated that m6A reader protein YTHDF5-mediated anti-cancer potential of NK cells primarily depends on IL-5 stimulation to sustain NK cell survival and expansion [162]. Moreover, a recent work by Peng et al. showed that down-regulation of NK cell-derived IFN*γ* and TNF was positively associated with short survival of patients with gastric cancer [163]. Furthermore, a study in hepatocellular carcinoma also suggested that significantly decreased production of IFN*γ* due to fewer NK cell within tumor was responsible for poor survival [164]. Finally, we also found the anti-cancer effect of NK cell was mediated by the secretion of IFNβ [165]. Interestingly, research has also found NK cells can reduce their IFN*γ* production via up-regulation of CTLA4, which is accompanied by tumor development, thereby promoting tumor progression [166]. Similarly, Thacker et al. revealed breast cancer stem cells were activated by NK cells; however, abrogation NK or blocking NK-secreted Wnt prevented breast cancer progression [167]. These results indicate that NK cell plays both pro- and anti-cancer in tumor evolution, and the tumor stage controls specific tumor-promoting or tumor-suppressing function.

It has long been known neutrophils are the most abundant cells in the blood, meaning neutrophils may play conflicting roles in disease, particularly in cancer. As anticipated, a large body of research has unmasked mechanisms by which neutrophils fight cancer cells through secretions. For instance, IFNβ and IFN*γ* are two significant factors in TME that have been found to enhance the cytotoxicity of neutrophils, enabling them to combat early-stage breast cancer, lung cancer, and melanoma in both mouse and clinical models [168–170]. In addition to IFNβ and IFN*γ*, an investigation of early-stage lung cancer samples also found that GM-CSF is another enhancement factor that promotes neutrophils’ anti-cancer capacity [170]. Furthermore, a growing number of evidence supports gasotransmitter NO can cause cancer cell apoptosis. Finisguerra et al., using a mouse modle, found NO produced by neutrophils can directly induce cancer cell death, and this cytotoxicity depends upon the expression of the HGF receptor, suggesting HGF receptor may be the ‘Achilles’ heel’ of cancer [171]. Additionally, Lev Becker’s group recently studied the role of neutrophils in several types of cancer cells, including ovarian, breast, lung, prostate cancer and melanoma cells and found that neutrophil-released specific protease ELANE induces cancer cell apoptosis via elevated levels of ROS and activation of CD8^+^ T-cells, suggesting neutrophils may possess a broad anti-cancer function [172]. However, clinical data suggests a high number of neutrophils in patients with advanced cancer is often associated with poor survival, indicating neutrophils may act as an accessory to the mastermind to assist tumor development. A great deal of study has been conducted to support this and answer how neutrophils corrupt to conspire to crime. For example, the roles of HGF and ROS, secreted from neutrophils, were examined, and their promotion role in tumor evolution was verified [171, 173], further suggesting neutrophil plasticity could be co-opted as a breakthrough in tumor therapy. This may be due to the higher concentration of PGE2 and TGFβ in late-stage, as well as other players in TME that have been demonstrated to accelerate tumor aggressiveness [174, 175]. Importantly, the production of PGE2 and TGFβ can both stem from cancer cells and non-canner cells, creating a reciprocal vicious cycle that promotes tumor progression. Unlike Lev Becker’s report, Maas et al. attempted to figure out whether neutrophils likely play the same role in brain tumor as they do in non-brain tumor, acting both pro- and anti-cancer via ROS. They found that ROS derived from neutrophils was decreased by glyoxalase 1 and IL19 to prolong neutrophile survival to prevent glioma cell from death [176]. The rational explanation is that the abundance and diversity of the components in the brain TME are less than their non-brain TME counterpart. Another good example of neutrophil pro-tumor is via inflammatory factor IL17a. More recently, Khalid et al. unveiled that increased IL17a relates to short survival. Further study manifested that the pro-tumor function of IL17a was exerted by recruiting neutrophils [177], strongly reflecting neutrophils played a key role in promoting cancer. Together, these data suggest neutrophils play a contradictory role during tumor evolution, as illustrated in Fig. 4. Exploring how to convert the pro-tumorigenic role to a tumor-suppressive one is a hotspot field in future investigation.

## Endothelial cells and tumor cell EMT activation

Blood vessels transport nutrients and oxygen to satisfy every cell’s demand, deliver paracrine factors, for instance, VEGF and Wnt, and take away metabolic waste such as lactic acid to maintain cell homeostasis. Compared with normal cells, cancer cells possess the capability of limitless proliferation; hence, they have to evolve abundant vascular branches to obtain more nutrients, oxygen, and vast growth factors and/or cytokines [178]. Angiogenesis, also termed as blood vessel growth, is the course endothelial cells (ECs) take advantage of pre-existing blood vessel to form new branched blood vessels and is one of the six acquired hallmarks of cancer cell, playing a crucial role in the development, organ homeostasis, tissue repair, as well as tumorigenesis and progression [9, 179].

So far, accumulating evidence has suggested that ECs have been involved both in normal and tumor cell EMT activation in a paracrine manner. For example, reports showed that liver and pancreatic organogenesis were closely associated with ECs-promoted EMT activation. Importantly, this facilitation is mediated by the ECs-produced and secreted VEGF (a prime player in blood vessel formation) [180, 181]. Moreover, the co-culture of ECs with breast cancer cells leads to EMT-controlled cancer cell migration, invasion, and metastasis, which were enhanced when CCL5, from ECs, binds to CCR5, a chemokine receptor of cancer cells [182]. Furthermore, data show that ECs promoted EMT of cervical cancer cell via Notch1/Lox/Snail pathway, which may be determined by the Dll4 secretion from ECs [179, 183]. Further studies are needed to figure out the detailed connection between ECs and tumor cell EMT-induction.

## Therapeutic targeting of EMT inducers

Considering the significance of secretions that can induce EMT and act either as a tumor suppressive or a pro-tumorigenesis in early- and late-stage (see in Fig. 4), respectively, it attracts considerable interest to develop new drugs and explore therapeutic avenues to triumph in the war between survival and cancer-related death. As for therapeutic approaches, surgery is the optimal choice and the markedly curative treatment for patients with solid tumor; however, tumor recurrence will inevitably be observed a few years or even months after surgical removal. Radiochemotherapy becomes the sole life-saving option for patients with advanced-stage inoperable disease.

Unfortunately, despite huge efforts and the approval of various drugs, cancer remains the leading cause that seriously threatens patients’ survival. Here, we briefly recapitulate the agents that have been utilized to kill EMT-fueled cancer cells, as summarized in Table 1. For example, the Netrin-1 antibody NP137 has been demonstrated to block both endometrial, skin squamous cell, and lung cancer cell growth via inhibiting EMT (NCT02977195 (Ref. [184, 185])). Moreover, bevacizumab, a monoclonal antibody of VEGF, is widely used in patients with solid tumor such as CRC and NSCLC to improve survival [186, 187]. Furthermore, in order to overcome the pro-tumoral role of Wnt in late-stage tumor, OMP-54F28, G007-LK, and SFRP-2 were administered [188–190].

**Table 1:** Therapy directed against part of EMT inducers

Targets	Drugs	Cancer types	References
COX2	Celecoxib, rofecoxib	CRC	[191–193]
CXCL5	SB225002, SB265610	LCa, CRC	[194, 195]
CXCL10/11	AMG487	CRC, BCa	[196, 197]
EGF	Lapatinib, panitumumab, gefitinib	BCa, CRC, LCa, NSCLC	[198–203]
HGF	AMG102, cabozantinib	EWS, PCa, HCC	[204–206]
GM-CSF	Lenzilumab, sipuleucel-T, T-VEC	ML, PCa, MM	[207–209]
IL6	Tocilizumab, siltuximab	BCa, LCa, OCa, PCa	[210–213]
IL10	αIL-10	CRC, OCa	[214, 215]
IFNβ	IFNAR1 mAb	MM	[216]
Netrin-1	NP137	ECs, SCC, NSCLC	[184, 185]
PGE2	SC58236, AH23848	BCa, CRC	[217, 218]
STAT3	AG490, WP1066	CRC, MM, GBM, HNSCC	[219–222]
TGFβ	LY2157299, SB505124, galunisertib	GCa, GBM, PDA	[223–226]
VEGF	Bevacizumab, ramucirumab, Sorafenib	CRC, NSCLC, GCa	[186, 187, 227–229]
Wnt	Aspirin, ipafricept, OMP-54F28, G007-LK, SFRP-2	CRC, PDA, GCT, HCC, PCa	[188–190, 230, 231]

*BCa* breast carcinoma, *CRC* colorectal cancer, *ECs* endometrial carcinomas, *EWS* Ewing sarcoma, *HCC* hepatocellular carcinoma, *GCT* germ cell tumor, *GBM* glioblastoma cancer, *GCa* gastric cancer, *HNSCC* head and neck squamous cell cancer, *LCa* lung carcinoma, *ML* myeloid leukemia, *MM* malignant melanoma, *NSCLC* Non-small-cell lung carcinoma, *OCa* ovarian carcinoma, *PDA* pancreatic ductal adenocarcinoma, *SCC* skin squamous cell carcinoma

Drug-mediated chemotherapy and immunotherapy are major weapons in our armory to fight against aberrant transforming cells. These drugs include monoclonal antibodies (for example, NP137 and Bevacizumab), small molecule inhibitors such as OMP-54F28, and inhibitory proteins (for instance, SFRP-2), which enriched our personal treatment modalities and will also illuminate the ways forward in the night sky as we strive to combat cancer.

## Conclusions and perspectives

**Fig. 5:**
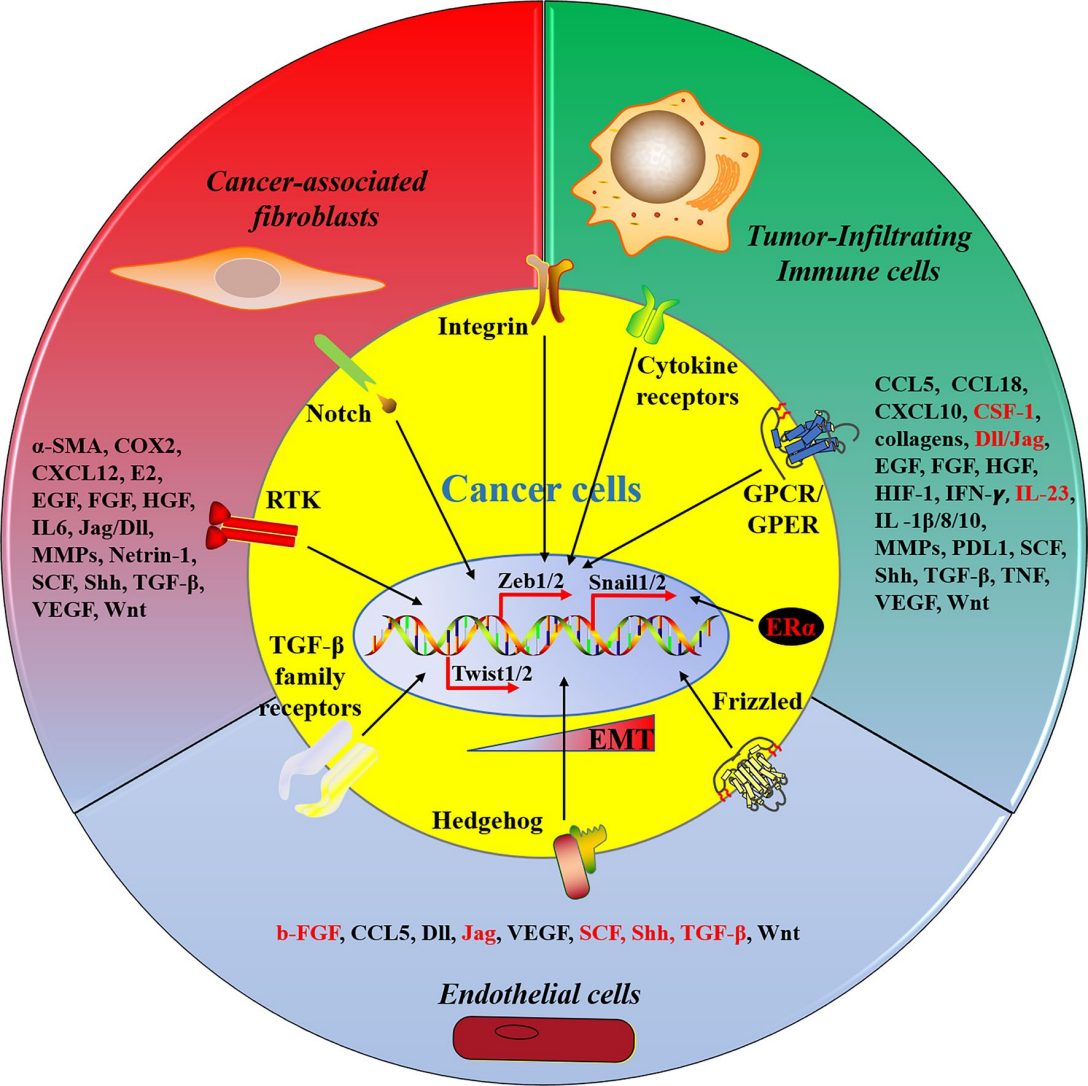
Summary of major stromal cells supporting tumor cell EMT via their secretions. Tumor microenvironments contain three main types of stromal cells that secrete diverse signal molecules. These secreted molecules, such as we enumerated, initiate tumor cell EMT programmes in a paracrine manner via binding to their receptors. For example, both E2 and chemokines can directly bind to GRCR/GPER of cancer cells to fuel EMT. Additionally, E2 can also stimulate tumor cell EMT through ERα activation. Cytokines, growth factors, and components of ECM facilitate tumor cell EMT by cytokine receptors signalling, TGF-β, RTK, and Integrin pathways, respectively. Notably, the marked secretions (red) listed here have either been shown elsewhere to promote tumor cell EMT, or at least there is currently no direct evidence to support tumor cell EMT activation

In summary, stromal cells significantly promote tumor growth by triggering EMT through paracrine signaling in the localized tumor microenvironment. They secrete a variety of cytokines that initiate signaling pathways involved in EMT, affecting the expression of EMT-TFs. This complex network of EMT signaling results from the synergistic interaction of cytokines and signaling pathways. Figure 5 provides a schematic representation of the stromal cells discussed in this article, illustrating their secretion of various bioproducts that regulate EMT. The use of the association between stromal cells and EMT for intervening in tumor initiation and progression has only recently been explored. Further research on stromal cells will contribute to a deeper understanding of the mechanisms underlying tumor formation and progression, the discovery of novel biomarkers for early cancer diagnosis, and the identification of potential treatment targets for malignant diseases.

Although we have made great achievements in cancer research and tumor treatment, there are unsurprisingly several unresolved issues in the research of the link between stromal cells and tumor cell EMT at present:

1. The biological network of the specific signaling molecules and pathways of EMT induced by stromal cells needs to be further improved;
2. The specific markers for diagnosing and treating stromal cells associated with EMT have not been clearly defined;
3. The molecular mechanisms that various stromal cell-induced EMT-TFs regulate the EMT program remain unclear;
4. Whether excretions from tumor-associated stromal cells, can induce EMT in cancer cell only as described in Table 2 remain unknown.

**Table 2:** Stromal cell-derived secretions influence EMT

Cell types	Sub-types	Secretions	Cancer types	References
CAFs		COX2, E2, EGF, FGF, HGF, IL-6, Jag/Dll, Wnt, MMPs, Netrin-1, Shh, TGF-β, VEGF, α-SMA, CXCL-12	BCa, GCa, OCa, NSCLC, PCa, PAAD, CRC, MM	[108, 111, 113, 115–120, 232–235]
TIICs	TAMs	TGF-β, IL-1β/6/10, HIF-1, CXCL10, VEGF, Wnt, TNF, CCL18	BCa, CRC, HCC, LCa, THCa	[137–140, 144–148]
	MDSCs	COX2, CCL11, EGF, HGF, CXCL12, TNF, collagen, IL-6, CCL2	BCa, CRC, NPC, Oca, NSCLC	[150–152, 154–156]
	CD8^+^ T cells	PD-L1, IFNγ, TGF-β and TNF-α	ESCC, BCa, CRC	[146, 157, 158]
	CD4^+^ T cells	TGF-β, TNF, IL-6, VEGF	BCa, PDAC, CRC	[161, 236, 237]
	NK cells	IL-5, IFNγ, TNF, IFNβ, GM-CSF	MM, GCa, HCC, BCa, NPC	[162–167]
	Nertrophils	IFNβ, IFNγ, NO, HGF, ELANE, ROS, PGE2, TGFβ, IL-17a	BCa, LCa, MM, Oca, BCa, CRC, GBM	[168–177]
ECs	Tip cells	VEGF, Wnt, CCL5, Dll4	BCa, CCa	[178, 182, 183]

*CCa* cervical cancer, *ESCC* esophageal squamous cell carcinoma, *NPC* nasopharyngeal cancer, *THCa* thyroid carcinoma, *TIIC* tumor-infiltrating immune cells

Similarly, the process of EMT involves a dynamic interplay between epithelial and mesenchymal cells, resulting in reciprocal alterations. Tumor cells exhibiting characteristics reminiscent of mesenchymal cells include a notable propensity for mobility, invasiveness, immune system avoidance, and resistance to chemotherapy. While the precise impact of compromised stromal cells on the potency of EMT remains somewhat ambiguous, it is evident that stromal cells release cytokines that prompt EMT, thereby augmenting the invasive and metastatic capabilities of epithelial cancer cells, bolstering immune resistance, and facilitating the acquisition of mesenchymal cell traits. Despite the precise method by which stromal cells in the tumor microenvironment initiate EMT is not fully understood, their importance in this process has been widely recognized. Hence, if one were to consider all of the stromal cells, including those previously mentioned and those not yet described, as active participants rather than passive observers and subject them to thorough analysis in order to mitigate, to some extent, the adverse effects of targeted therapy, it is logically justifiable to have confidence in the potential for substantial progress in the advancement of medications that target peripheral stromal cells. This progress could potentially lead to the prevention of tumor metastasis and, ultimately, the eradication of cancer in the foreseeable future.

## Data Availability

Not applicable.

## Abbreviations

AR: Androgen receptor
α-SMA: α-Smooth muscle actin
CAFs: Cancer-associated fibroblasts
CSCs: Cancer stem cells
CTCs: Circulating tumor cells
CTLA4: Cytotoxic T lymphocyte-associated protein 4
DCC: Deleted in colorectal carcinoma
DTCs: Disseminated tumor cells
E2: 17 β-Estradiol
ECM: Extracellular matrix
ECs: Endothelial cells
EMT: Epithelial–mesenchymal transition
EMT-TFs: EMT-induced transcription factors
ERα: Estrogen receptor α
FAK: Focal adhesion kinase
Fsp1: Fibroblast specific protein 1
GC-MSC: Gastric cancer-derived mesenchymal stromal cells
GPCR: G protein coupled receptors
GPER: G protein coupled estrogen receptor
GR: Glucocorticoid receptor
HGF: Hepatocyte growth factor
HPMCs: Human peritoneal mesothelial cells
IL-6: Interleukin 6
MDSCs: Marrow-derived suppressor cells
MET: Mesenchymal–epithelial transition
MMPs: Matrix metalloproteinases
MRD: Minimal residual disease
NSCLC: Non-small cell lung cancer
PD1: Programmed-cell death protein 1
PD-L1/2: Programmed-cell death ligand 1/2
PDGF: Platelet-derived growth factor
PI3K: Phosphoinositide-3-kinase
RTK: Receptor tyrosine kinase
SDF1: Stromal cell-derived factor-1
Shh: Sonic Hedgehog
TAMs: Tumor-associated macrophages
TGF-β: Transforming growth factor β
TICs: Tumour-initiating cells
TIME: Tumor immune microenvironment
TIICs: Tumor-infiltrating immune cells
TMEs: Tumor microenvironments
TNF: Tumor necrosis factor
T-regs: Regulatory T cells
VEGF: Vascular endothelial growth factor

## References

[1] Gupta GP, Massague J (2006). Cancer metastasis: building a framework. Cell.

[2] Chaffer CL, Weinberg RA (2011). A perspective on cancer cell metastasis. Science.

[3] Ruan W, Yuan X, Eltzschig HK (2021). Circadian rhythm as a therapeutic target. Nat Rev Drug Discov.

[4] Bishehsari F, Voigt RM, Keshavarzian A (2020). Circadian rhythms and the gut microbiota: from the metabolic syndrome to cancer. Nat Rev Endocrinol.

[5] Hanahan D, Weinberg RA (2011). Hallmarks of cancer: the next generation. Cell.

[6] Robinson D, Van Allen EM, Wu YM, Schultz N, Lonigro RJ, Mosquera JM, Montgomery B, Taplin ME, Pritchard CC, Attard G (2015). Integrative clinical genomics of advanced prostate cancer. Cell.

[7] Lu Z, Zou J, Li S, Topper MJ, Tao Y, Zhang H, Jiao X, Xie W, Kong X, Vaz M (2020). Epigenetic therapy inhibits metastases by disrupting premetastatic niches. Nature.

[8] Faubert B, Solmonson A, DeBerardinis RJ (2020). Metabolic reprogramming and cancer progression. Science.

[9] Hanahan D (2022). Hallmarks of cancer: new dimensions. Cancer Discov.

[10] Gupta PB, Pastushenko I, Skibinski A, Blanpain C, Kuperwasser C (2019). Phenotypic plasticity: driver of cancer initiation, progression, and therapy resistance. Cell Stem Cell.

[11] Boumahdi S, de Sauvage FJ (2020). The great escape: tumour cell plasticity in resistance to targeted therapy. Nat Rev Drug Discov.

[12] Sung H, Ferlay J, Siegel RL, Laversanne M, Soerjomataram I, Jemal A, Bray F (2021). Global cancer statistics 2020: GLOBOCAN estimates of incidence and mortality worldwide for 36 cancers in 185 countries. CA Cancer J Clin.

[13] Quintanal-Villalonga A, Chan JM, Yu HA, Pe'er D, Sawyers CL, Sen T, Rudin CM (2020). Lineage plasticity in cancer: a shared pathway of therapeutic resistance. Nat Rev Clin Oncol.

[14] Ku SY, Rosario S, Wang Y, Mu P, Seshadri M, Goodrich ZW, Goodrich MM, Labbe DP, Gomez EC, Wang J (2017). Rb1 and Trp53 cooperate to suppress prostate cancer lineage plasticity, metastasis, and antiandrogen resistance. Science.

[15] Mu P, Zhang Z, Benelli M, Karthaus WR, Hoover E, Chen CC, Wongvipat J, Ku SY, Gao D, Cao Z (2017). SOX2 promotes lineage plasticity and antiandrogen resistance in TP53- and RB1-deficient prostate cancer. Science.

[16] Lin SC, Chou YT, Jiang SS, Chang JL, Chung CH, Kao YR, Chang IS, Wu CW (2016). Epigenetic switch between SOX2 and SOX9 regulates cancer cell plasticity. Cancer Res.

[17] Davies AH, Beltran H, Zoubeidi A (2018). Cellular plasticity and the neuroendocrine phenotype in prostate cancer. Nat Rev Urol.

[18] Mayayo-Peralta I, Prekovic S, Zwart W (2021). Estrogen receptor on the move: cistromic plasticity and its implications in breast cancer. Mol Aspects Med.

[19] Roswall P, Bocci M, Bartoschek M, Li H, Kristiansen G, Jansson S, Lehn S, Sjolund J, Reid S, Larsson C (2018). Microenvironmental control of breast cancer subtype elicited through paracrine platelet-derived growth factor-CC signaling. Nat Med.

[20] Chaffer CL, San Juan BP, Lim E, Weinberg RA (2016). EMT, cell plasticity and metastasis. Cancer Metastasis Rev.

[21] Wilson MM, Weinberg RA, Lees JA, Guen VJ (2020). Emerging mechanisms by which EMT programs control stemness. Trends Cancer.

[22] Nieto MA, Huang RY, Jackson RA, Thiery JP (2016). Emt: 2016. Cell.

[23] Brabletz T, Kalluri R, Nieto MA, Weinberg RA (2018). EMT in cancer. Nat Rev Cancer.

[24] Lambert AW, Weinberg RA (2021). Linking EMT programmes to normal and neoplastic epithelial stem cells. Nat Rev Cancer.

[25] Thiery JP, Acloque H, Huang RY, Nieto MA (2009). Epithelial–mesenchymal transitions in development and disease. Cell.

[26] Kar R, Jha NK, Jha SK, Sharma A, Dholpuria S, Asthana N, Chaurasiya K, Singh VK, Burgee S, Nand P (2019). A “NOTCH” deeper into the epithelial-to-mesenchymal transition (EMT) program in breast cancer. Genes.

[27] Pei D, Shu X, Gassama-Diagne A, Thiery JP (2019). Mesenchymal–epithelial transition in development and reprogramming. Nat Cell Biol.

[28] Lu W, Kang Y (2019). Epithelial–mesenchymal plasticity in cancer progression and metastasis. Dev Cell.

[29] Pastushenko I, Brisebarre A, Sifrim A, Fioramonti M, Revenco T, Boumahdi S, Van Keymeulen A, Brown D, Moers V, Lemaire S (2018). Identification of the tumour transition states occurring during EMT. Nature.

[30] Brabletz S, Schuhwerk H, Brabletz T, Stemmler MP (2021). Dynamic EMT: a multi-tool for tumor progression. EMBO J.

[31] Shibue T, Weinberg RA (2017). EMT, CSCs, and drug resistance: the mechanistic link and clinical implications. Nat Rev Clin Oncol.

[32] Bakir B, Chiarella AM, Pitarresi JR, Rustgi AK (2020). EMT, MET, plasticity, and tumor metastasis. Trends Cell Biol.

[33] Loo SY, Toh LP, Xie WH, Pathak E, Tan W, Ma S, Lee MY, Shatishwaran S, Yeo JZZ, Yuan J (2021). Fatty acid oxidation is a druggable gateway regulating cellular plasticity for driving metastasis in breast cancer. Sci Adv.

[34] Ognjenovic NB, Bagheri M, Mohamed GA, Xu K, Chen Y, Mohamed Saleem MA, Brown MS, Nagaraj SH, Muller KE, Gerber SA (2020). Limiting self-renewal of the basal compartment by PKA activation induces differentiation and alters the evolution of mammary tumors. Dev Cell.

[35] Pattabiraman DR, Bierie B, Kober KI, Thiru P, Krall JA, Zill C, Reinhardt F, Tam WL, Weinberg RA (2016). Activation of PKA leads to mesenchymal-to-epithelial transition and loss of tumor-initiating ability. Science.

[36] Zhang N, Ng AS, Cai S, Li Q, Yang L, Kerr D (2021). Novel therapeutic strategies: targeting epithelial–mesenchymal transition in colorectal cancer. Lancet Oncol.

[37] Taki M, Abiko K, Ukita M, Murakami R, Yamanoi K, Yamaguchi K, Hamanishi J, Baba T, Matsumura N, Mandai M (2021). Tumor immune microenvironment during epithelial–mesenchymal transition. Clin Cancer Res.

[38] Dongre A, Weinberg RA (2019). New insights into the mechanisms of epithelial–mesenchymal transition and implications for cancer. Nat Rev Mol Cell Biol.

[39] Migita T, Ueda A, Ohishi T, Hatano M, Seimiya H, Horiguchi SI, Koga F, Shibasaki F (2017). Epithelial–mesenchymal transition promotes SOX2 and NANOG expression in bladder cancer. Lab Invest.

[40] Mahmood MQ, Ward C, Muller HK, Sohal SS, Walters EH (2017). Epithelial mesenchymal transition (EMT) and non-small cell lung cancer (NSCLC): a mutual association with airway disease. Med Oncol.

[41] Chen T, You Y, Jiang H, Wang ZZ (2017). Epithelial–mesenchymal transition (EMT): a biological process in the development, stem cell differentiation, and tumorigenesis. J Cell Physiol.

[42] Stemmler MP, Eccles RL, Brabletz S, Brabletz T (2019). Non-redundant functions of EMT transcription factors. Nat Cell Biol.

[43] Imani S, Hosseinifard H, Cheng J, Wei C, Fu J (2016). Prognostic value of EMT-inducing transcription factors (EMT-TFs) in metastatic breast cancer: a systematic review and meta-analysis. Sci Rep.

[44] Medici D, Nawshad A (2010). Type I collagen promotes epithelial–mesenchymal transition through ILK-dependent activation of NF-kappaB and LEF-1. Matrix Biol.

[45] Kahlert C, Lahes S, Radhakrishnan P, Dutta S, Mogler C, Herpel E, Brand K, Steinert G, Schneider M, Mollenhauer M (2011). Overexpression of ZEB2 at the invasion front of colorectal cancer is an independent prognostic marker and regulates tumor invasion in vitro. Clin Cancer Res.

[46] Qi LN, Xiang BD, Wu FX, Ye JZ, Zhong JH, Wang YY, Chen YY, Chen ZS, Ma L, Chen J (2018). Circulating tumor cells undergoing EMT provide a metric for diagnosis and prognosis of patients with hepatocellular carcinoma. Cancer Res.

[47] Li H, Li J, Chen L, Qi S, Yu S, Weng Z, Hu Z, Zhou Q, Xin Z, Shi L (2019). HERC3-mediated SMAD7 ubiquitination degradation promotes autophagy-induced EMT and chemoresistance in glioblastoma. Clin Cancer Res.

[48] Steinbichler TB, Dudas J, Skvortsov S, Ganswindt U, Riechelmann H, Skvortsova II (2018). Therapy resistance mediated by cancer stem cells. Semin Cancer Biol.

[49] Lu S, Yu L, Mu Y, Ma J, Tian J, Xu W, Wang H (2014). Role and mechanism of Twist1 in modulating the chemosensitivity of FaDu cells. Mol Med Rep.

[50] Hsu DS, Lan HY, Huang CH, Tai SK, Chang SY, Tsai TL, Chang CC, Tzeng CH, Wu KJ, Kao JY (2010). Regulation of excision repair cross-complementation group 1 by Snail contributes to cisplatin resistance in head and neck cancer. Clin Cancer Res.

[51] Zheng X, Carstens JL, Kim J, Scheible M, Kaye J, Sugimoto H, Wu CC, LeBleu VS, Kalluri R (2015). Epithelial-to-mesenchymal transition is dispensable for metastasis but induces chemoresistance in pancreatic cancer. Nature.

[52] Oskarsson T, Batlle E, Massague J (2014). Metastatic stem cells: sources, niches, and vital pathways. Cell Stem Cell.

[53] Massague J, Obenauf AC (2016). Metastatic colonization by circulating tumour cells. Nature.

[54] Najafi M, Mortezaee K, Majidpoor J (2019). Cancer stem cell (CSC) resistance drivers. Life Sci.

[55] Rambow F, Rogiers A, Marin-Bejar O, Aibar S, Femel J, Dewaele M, Karras P, Brown D, Chang YH, Debiec-Rychter M (2018). Toward minimal residual disease-directed therapy in melanoma. Cell.

[56] Lytle NK, Barber AG, Reya T (2018). Stem cell fate in cancer growth, progression and therapy resistance. Nat Rev Cancer.

[57] Mendonsa AM, Na TY, Gumbiner BM (2018). E-cadherin in contact inhibition and cancer. Oncogene.

[58] Li S, Xu F, Zhang J, Wang L, Zheng Y, Wu X, Wang J, Huang Q, Lai M (2018). Tumor-associated macrophages remodeling EMT and predicting survival in colorectal carcinoma. Oncoimmunology.

[59] Kalluri R, Weinberg RA (2009). The basics of epithelial–mesenchymal transition. J Clin Invest.

[60] Lin S, Taylor MD, Singh PK, Yang S (2021). How does fascin promote cancer metastasis?. FEBS J.

[61] Poli G, Ruggiero C, Cantini G, Canu L, Baroni G, Armignacco R, Jouinot A, Santi R, Ercolino T, Ragazzon B (2019). Fascin-1 is a novel prognostic biomarker associated with tumor invasiveness in adrenocortical carcinoma. J Clin Endocrinol Metab.

[62] Chen Y, LeBleu VS, Carstens JL, Sugimoto H, Zheng X, Malasi S, Saur D, Kalluri R (2018). Dual reporter genetic mouse models of pancreatic cancer identify an epithelial-to-mesenchymal transition-independent metastasis program. EMBO Mol Med.

[63] Nakatani K, Asai O, Konishi N, Iwano M (2021). Role of fibroblast specific protein 1 expression in the progression of adriamycin-induced glomerulosclerosis. Biochem Biophys Res Commun.

[64] Saitoh M (2018). Involvement of partial EMT in cancer progression. J Biochem.

[65] Nantajit D, Lin D, Li JJ (2015). The network of epithelial–mesenchymal transition: potential new targets for tumor resistance. J Cancer Res Clin Oncol.

[66] Katsuno Y, Meyer DS, Zhang Z, Shokat KM, Akhurst RJ, Miyazono K, Derynck R (2019). Chronic TGF-beta exposure drives stabilized EMT, tumor stemness, and cancer drug resistance with vulnerability to bitopic mTOR inhibition. Sci Signal.

[67] Lee HM, Hwang KA, Choi KC (2017). Diverse pathways of epithelial mesenchymal transition related with cancer progression and metastasis and potential effects of endocrine disrupting chemicals on epithelial mesenchymal transition process. Mol Cell Endocrinol.

[68] Yang J, Antin P, Berx G, Blanpain C, Brabletz T, Bronner M, Campbell K, Cano A, Casanova J, Christofori G (2020). Guidelines and definitions for research on epithelial–mesenchymal transition. Nat Rev Mol Cell Biol.

[69] Valastyan S, Weinberg RA (2011). Tumor metastasis: molecular insights and evolving paradigms. Cell.

[70] Klein CA (2009). Parallel progression of primary tumours and metastases. Nat Rev Cancer.

[71] Dongre A, Rashidian M, Reinhardt F, Bagnato A, Keckesova Z, Ploegh HL, Weinberg RA (2017). Epithelial-to-mesenchymal transition contributes to immunosuppression in breast carcinomas. Cancer Res.

[72] Ren T, Zheng B, Huang Y, Wang S, Bao X, Liu K, Guo W (2019). Osteosarcoma cell intrinsic PD-L2 signals promote invasion and metastasis via the RhoA-ROCK-LIMK2 and autophagy pathways. Cell Death Dis.

[73] Jiang Y, Zhan H (2020). Communication between EMT and PD-L1 signaling: new insights into tumor immune evasion. Cancer Lett.

[74] Kontos F, Michelakos T, Kurokawa T, Sadagopan A, Schwab JH, Ferrone CR, Ferrone S (2021). B7-H3: an attractive target for antibody-based immunotherapy. Clin Cancer Res.

[75] Paget S (1989). The distribution of secondary growths in cancer of the breast. Cancer Metastasis Rev.

[76] Weiss L (1992). Comments on hematogenous metastatic patterns in humans as revealed by autopsy. Clin Exp Metastasis.

[77] Gao Y, Bado I, Wang H, Zhang W, Rosen JM, Zhang XH (2019). Metastasis organotropism: redefining the congenial soil. Dev Cell.

[78] Jin X, Demere Z, Nair K, Ali A, Ferraro GB, Natoli T, Deik A, Petronio L, Tang AA, Zhu C (2020). A metastasis map of human cancer cell lines. Nature.

[79] DeBerardinis RJ (2020). Tumor microenvironment, metabolism, and immunotherapy. N Engl J Med.

[80] Bader JE, Voss K, Rathmell JC (2020). Targeting metabolism to improve the tumor microenvironment for cancer immunotherapy. Mol Cell.

[81] Wei C, Yang C, Wang S, Shi D, Zhang C, Lin X, Liu Q, Dou R, Xiong B (2019). Crosstalk between cancer cells and tumor associated macrophages is required for mesenchymal circulating tumor cell-mediated colorectal cancer metastasis. Mol Cancer.

[82] Cowman SJ, Fuja DG, Liu XD, Tidwell RSS, Kandula N, Sirohi D, Agarwal AM, Emerson LL, Tripp SR, Mohlman JS (2020). Macrophage HIF-1alpha is an independent prognostic indicator in kidney cancer. Clin Cancer Res.

[83] MacKie RM, Reid R, Junor B (2003). Fatal melanoma transferred in a donated kidney 16 years after melanoma surgery. N Engl J Med.

[84] Matser YAH, Terpstra ML, Nadalin S, Nossent GD, de Boer J, van Bemmel BC, van Eeden S, Budde K, Brakemeier S, Bemelman FJ (2018). Transmission of breast cancer by a single multiorgan donor to 4 transplant recipients. Am J Transplant.

[85] Morris-Stiff G, Steel A, Savage P, Devlin J, Griffiths D, Portman B, Mason M, Jurewicz WA, Welsh Transplantation Research G (2004). Transmission of donor melanoma to multiple organ transplant recipients. Am J Transplant.

[86] Castets M, Broutier L, Molin Y, Brevet M, Chazot G, Gadot N, Paquet A, Mazelin L, Jarrosson-Wuilleme L, Scoazec JY (2011). DCC constrains tumour progression via its dependence receptor activity. Nature.

[87] Meurette O, Mehlen P (2018). Notch signaling in the tumor microenvironment. Cancer Cell.

[88] Wang H, Boussouar A, Mazelin L, Tauszig-Delamasure S, Sun Y, Goldschneider D, Paradisi A, Mehlen P (2018). The proto-oncogene c-kit inhibits tumor growth by behaving as a dependence receptor. Mol Cell.

[89] Gibert B, Mehlen P (2015). Dependence receptors and cancer: addiction to trophic ligands. Cancer Res.

[90] Chen X, Song E (2019). Turning foes to friends: targeting cancer-associated fibroblasts. Nat Rev Drug Discov.

[91] Chen Y, McAndrews KM, Kalluri R (2021). Clinical and therapeutic relevance of cancer-associated fibroblasts. Nat Rev Clin Oncol.

[92] Sahai E, Astsaturov I, Cukierman E, DeNardo DG, Egeblad M, Evans RM, Fearon D, Greten FR, Hingorani SR, Hunter T (2020). A framework for advancing our understanding of cancer-associated fibroblasts. Nat Rev Cancer.

[93] Gascard P, Tlsty TD (2016). Carcinoma-associated fibroblasts: orchestrating the composition of malignancy. Genes Dev.

[94] Mosa MH, Michels BE, Menche C, Nicolas AM, Darvishi T, Greten FR, Farin HF (2020). A Wnt-induced phenotypic switch in cancer-associated fibroblasts inhibits EMT in colorectal cancer. Cancer Res.

[95] Ren Y, Jia HH, Xu YQ, Zhou X, Zhao XH, Wang YF, Song X, Zhu ZY, Sun T, Dou Y (2018). Paracrine and epigenetic control of CAF-induced metastasis: the role of HOTAIR stimulated by TGF-ss1 secretion. Mol Cancer.

[96] Wang Y, Jing Y, Ding L, Zhang X, Song Y, Chen S, Zhao X, Huang X, Pu Y, Wang Z (2019). Epiregulin reprograms cancer-associated fibroblasts and facilitates oral squamous cell carcinoma invasion via JAK2-STAT3 pathway. J Exp Clin Cancer Res.

[97] Bhowmick NA, Chytil A, Plieth D, Gorska AE, Dumont N, Shappell S, Washington MK, Neilson EG, Moses HL (2004). TGF-beta signaling in fibroblasts modulates the oncogenic potential of adjacent epithelia. Science.

[98] Song M, He J, Pan QZ, Yang J, Zhao J, Zhang YJ, Huang Y, Tang Y, Wang Q, He J (2021). Cancer-associated fibroblast-mediated cellular crosstalk supports hepatocellular carcinoma progression. Hepatology.

[99] Kojima Y, Acar A, Eaton EN, Mellody KT, Scheel C, Ben-Porath I, Onder TT, Wang ZC, Richardson AL, Weinberg RA (2010). Autocrine TGF-beta and stromal cell-derived factor-1 (SDF-1) signaling drives the evolution of tumor-promoting mammary stromal myofibroblasts. Proc Natl Acad Sci USA.

[100] Colak S, Ten Dijke P (2017). Targeting TGF-beta signaling in cancer. Trends Cancer.

[101] Massague J (2012). TGFbeta signalling in context. Nat Rev Mol Cell Biol.

[102] Huynh LK, Hipolito CJ, Ten Dijke P (2019). A perspective on the development of TGF-beta inhibitors for cancer treatment. Biomolecules.

[103] Mu Y, Gudey SK, Landstrom M (2012). Non-Smad signaling pathways. Cell Tissue Res.

[104] Yoshida GJ (2020). Regulation of heterogeneous cancer-associated fibroblasts: the molecular pathology of activated signaling pathways. J Exp Clin Cancer Res.

[105] Zhuang J, Lu Q, Shen B, Huang X, Shen L, Zheng X, Huang R, Yan J, Guo H (2015). TGFbeta1 secreted by cancer-associated fibroblasts induces epithelial–mesenchymal transition of bladder cancer cells through lncRNA-ZEB2NAT. Sci Rep.

[106] Abubaker K, Luwor RB, Escalona R, McNally O, Quinn MA, Thompson EW, Findlay JK, Ahmed N (2014). Targeted disruption of the JAK2/STAT3 pathway in combination with systemic administration of paclitaxel inhibits the priming of ovarian cancer stem cells leading to a reduced tumor burden. Front Oncol.

[107] Shi Y, Tao M, Ni J, Tang L, Liu F, Chen H, Ma X, Hu Y, Zhou X, Qiu A (2021). Requirement of histone deacetylase 6 for interleukin-6 induced epithelial–mesenchymal transition, proliferation, and migration of peritoneal mesothelial cells. Front Pharmacol.

[108] Shintani Y, Fujiwara A, Kimura T, Kawamura T, Funaki S, Minami M, Okumura M (2016). IL-6 secreted from cancer-associated fibroblasts mediates chemoresistance in NSCLC by increasing epithelial–mesenchymal transition signaling. J Thorac Oncol.

[109] Wang L, Zhang F, Cui JY, Chen L, Chen YT, Liu BW (2018). CAFs enhance paclitaxel resistance by inducing EMT through the IL6/JAK2/STAT3 pathway. Oncol Rep.

[110] Wanami LS, Chen HY, Peiro S, Garcia de Herreros A, Bachelder RE (2008). Vascular endothelial growth factor-A stimulates Snail expression in breast tumor cells: implications for tumor progression. Exp Cell Res.

[111] Yang AD, Camp ER, Fan F, Shen L, Gray MJ, Liu W, Somcio R, Bauer TW, Wu Y, Hicklin DJ (2006). Vascular endothelial growth factor receptor-1 activation mediates epithelial to mesenchymal transition in human pancreatic carcinoma cells. Cancer Res.

[112] Masson V, de la Ballina LR, Munaut C, Wielockx B, Jost M, Maillard C, Blacher S, Bajou K, Itoh T, Itohara S (2005). Contribution of host MMP-2 and MMP-9 to promote tumor vascularization and invasion of malignant keratinocytes. FASEB J.

[113] Ding X, Ji J, Jiang J, Cai Q, Wang C, Shi M, Yu Y, Zhu Z, Zhang J (2018). HGF-mediated crosstalk between cancer-associated fibroblasts and MET-unamplified gastric cancer cells activates coordinated tumorigenesis and metastasis. Cell Death Dis.

[114] Comoglio PM, Trusolino L, Boccaccio C (2018). Known and novel roles of the MET oncogene in cancer: a coherent approach to targeted therapy. Nat Rev Cancer.

[115] Walter K, Omura N, Hong SM, Griffith M, Vincent A, Borges M, Goggins M (2010). Overexpression of smoothened activates the sonic hedgehog signaling pathway in pancreatic cancer-associated fibroblasts. Clin Cancer Res.

[116] Sung PJ, Rama N, Imbach J, Fiore S, Ducarouge B, Neves D, Chen HW, Bernard D, Yang PC, Bernet A (2019). Cancer-associated fibroblasts produce netrin-1 to control cancer cell plasticity. Cancer Res.

[117] Sizemore GM, Balakrishnan S, Hammer AM, Thies KA, Trimboli AJ, Wallace JA, Sizemore ST, Kladney RD, Woelke SA, Yu L (2017). Stromal PTEN inhibits the expansion of mammary epithelial stem cells through Jagged-1. Oncogene.

[118] Unterleuthner D, Neuhold P, Schwarz K, Janker L, Neuditschko B, Nivarthi H, Crncec I, Kramer N, Unger C, Hengstschlager M (2020). Cancer-associated fibroblast-derived WNT2 increases tumor angiogenesis in colon cancer. Angiogenesis.

[119] Santen RJ, Santner SJ, Pauley RJ, Tait L, Kaseta J, Demers LM, Hamilton C, Yue W, Wang JP (1997). Estrogen production via the aromatase enzyme in breast carcinoma: which cell type is responsible?. J Steroid Biochem Mol Biol.

[120] Soon PS, Kim E, Pon CK, Gill AJ, Moore K, Spillane AJ, Benn DE, Baxter RC (2013). Breast cancer-associated fibroblasts induce epithelial-to-mesenchymal transition in breast cancer cells. Endocr Relat Cancer.

[121] Iwai Y, Hamanishi J, Chamoto K, Honjo T (2017). Cancer immunotherapies targeting the PD-1 signaling pathway. J Biomed Sci.

[122] Sharma P, Wagner K, Wolchok JD, Allison JP (2011). Novel cancer immunotherapy agents with survival benefit: recent successes and next steps. Nat Rev Cancer.

[123] Binnewies M, Roberts EW, Kersten K, Chan V, Fearon DF, Merad M, Coussens LM, Gabrilovich DI, Ostrand-Rosenberg S, Hedrick CC (2018). Understanding the tumor immune microenvironment (TIME) for effective therapy. Nat Med.

[124] Tang F, Li J, Qi L, Liu D, Bo Y, Qin S, Miao Y, Yu K, Hou W, Li J (2023). A pan-cancer single-cell panorama of human natural killer cells. Cell.

[125] Bottcher JP, Bonavita E, Chakravarty P, Blees H, Cabeza-Cabrerizo M, Sammicheli S, Rogers NC, Sahai E, Zelenay S, Reis e Sousa C (2018). NK cells stimulate recruitment of cDC1 into the tumor microenvironment promoting cancer immune control. Cell.

[126] Jaillon S, Ponzetta A, Di Mitri D, Santoni A, Bonecchi R, Mantovani A (2020). Neutrophil diversity and plasticity in tumour progression and therapy. Nat Rev Cancer.

[127] Dinh HQ, Eggert T, Meyer MA, Zhu YP, Olingy CE, Llewellyn R, Wu R, Hedrick CC (2020). Coexpression of CD71 and CD117 identifies an early unipotent neutrophil progenitor population in human bone marrow. Immunity.

[128] Liu L, Hou Y, Deng C, Tao Z, Chen Z, Hu J, Chen K (2022). Single cell sequencing reveals that CD39 inhibition mediates changes to the tumor microenvironment. Nat Commun.

[129] DeNardo DG, Ruffell B (2019). Macrophages as regulators of tumour immunity and immunotherapy. Nat Rev Immunol.

[130] Mantovani A, Marchesi F, Malesci A, Laghi L, Allavena P (2017). Tumour-associated macrophages as treatment targets in oncology. Nat Rev Clin Oncol.

[131] Comito G, Giannoni E, Segura CP, Barcellos-de-Souza P, Raspollini MR, Baroni G, Lanciotti M, Serni S, Chiarugi P (2014). Cancer-associated fibroblasts and M2-polarized macrophages synergize during prostate carcinoma progression. Oncogene.

[132] Najafi M, Hashemi Goradel N, Farhood B, Salehi E, Nashtaei MS, Khanlarkhani N, Khezri Z, Majidpoor J, Abouzaripour M, Habibi M (2019). Macrophage polarity in cancer: a review. J Cell Biochem.

[133] Georgoudaki AM, Prokopec KE, Boura VF, Hellqvist E, Sohn S, Ostling J, Dahan R, Harris RA, Rantalainen M, Klevebring D (2016). Reprogramming tumor-associated macrophages by antibody targeting inhibits cancer progression and metastasis. Cell Rep.

[134] Zhan HX, Zhou B, Cheng YG, Xu JW, Wang L, Zhang GY, Hu SY (2017). Crosstalk between stromal cells and cancer cells in pancreatic cancer: new insights into stromal biology. Cancer Lett.

[135] Habtezion A, Edderkaoui M, Pandol SJ (2016). Macrophages and pancreatic ductal adenocarcinoma. Cancer Lett.

[136] Kloepper J, Riedemann L, Amoozgar Z, Seano G, Susek K, Yu V, Dalvie N, Amelung RL, Datta M, Song JW (2016). Ang-2/VEGF bispecific antibody reprograms macrophages and resident microglia to anti-tumor phenotype and prolongs glioblastoma survival. Proc Natl Acad Sci USA.

[137] Poh AR, Ernst M (2018). Targeting macrophages in cancer: from bench to bedside. Front Oncol.

[138] Zhang J, Zhang Q, Lou Y, Fu Q, Chen Q, Wei T, Yang J, Tang J, Wang J, Chen Y (2018). Hypoxia-inducible factor-1alpha/interleukin-1beta signaling enhances hepatoma epithelial–mesenchymal transition through macrophages in a hypoxic-inflammatory microenvironment. Hepatology.

[139] Cai J, Xia L, Li J, Ni S, Song H, Wu X (2019). Tumor-associated macrophages derived TGF-beta induced epithelial to mesenchymal transition in colorectal cancer cells through Smad 2,3-4/Snail signaling pathway. Cancer Res Treat.

[140] Dong F, Ruan S, Wang J, Xia Y, Le K, Xiao X, Hu T, Wang Q (2020). M2 macrophage-induced lncRNA PCAT6 facilitates tumorigenesis and angiogenesis of triple-negative breast cancer through modulation of VEGFR2. Cell Death Dis.

[141] Ye LY, Chen W, Bai XL, Xu XY, Zhang Q, Xia XF, Sun X, Li GG, Hu QD, Fu QH (2016). Hypoxia-induced epithelial-to-mesenchymal transition in hepatocellular carcinoma induces an immunosuppressive tumor microenvironment to promote metastasis. Cancer Res.

[142] Zhang Q, Zhang Y, Chen Y, Qian J, Zhang X, Yu K (2019). A novel mTORC1/2 inhibitor (MTI-31) inhibits tumor growth, epithelial–mesenchymal transition, metastases, and improves antitumor immunity in preclinical models of lung cancer. Clin Cancer Res.

[143] Li W, Zhang X, Wu F, Zhou Y, Bao Z, Li H, Zheng P, Zhao S (2019). Gastric cancer-derived mesenchymal stromal cells trigger M2 macrophage polarization that promotes metastasis and EMT in gastric cancer. Cell Death Dis.

[144] Jiang Y, Han Q, Zhao H, Zhang J (2021). Promotion of epithelial–mesenchymal transformation by hepatocellular carcinoma-educated macrophages through Wnt2b/beta-catenin/c-Myc signaling and reprogramming glycolysis. J Exp Clin Cancer Res.

[145] Lv J, Chen FK, Liu C, Liu PJ, Feng ZP, Jia L, Yang ZX, Hou F, Deng ZY (2020). Zoledronic acid inhibits thyroid cancer stemness and metastasis by repressing M2-like tumor-associated macrophages induced Wnt/beta-catenin pathway. Life Sci.

[146] Bates RC, Mercurio AM (2003). Tumor necrosis factor-alpha stimulates the epithelial-to-mesenchymal transition of human colonic organoids. Mol Biol Cell.

[147] Su S, Liu Q, Chen J, Chen J, Chen F, He C, Huang D, Wu W, Lin L, Huang W (2014). A positive feedback loop between mesenchymal-like cancer cells and macrophages is essential to breast cancer metastasis. Cancer Cell.

[148] Che D, Zhang S, Jing Z, Shang L, Jin S, Liu F, Shen J, Li Y, Hu J, Meng Q (2017). Macrophages induce EMT to promote invasion of lung cancer cells through the IL-6-mediated COX-2/PGE2/beta-catenin signalling pathway. Mol Immunol.

[149] De Cicco P, Ercolano G, Rubino V, Terrazzano G, Ruggiero G, Cirino G, Ianaro A (2020). Modulation of the functions of myeloid-derived suppressor cells : a new strategy of hydrogen sulfide anti-cancer effects. Br J Pharmacol.

[150] Lin S, Zhang X, Huang G, Cheng L, Lv J, Zheng D, Lin S, Wang S, Wu Q, Long Y (2021). Myeloid-derived suppressor cells promote lung cancer metastasis by CCL11 to activate ERK and AKT signaling and induce epithelial–mesenchymal transition in tumor cells. Oncogene.

[151] Sangaletti S, Tripodo C, Santangelo A, Castioni N, Portararo P, Gulino A, Botti L, Parenza M, Cappetti B, Orlandi R (2016). Mesenchymal transition of high-grade breast carcinomas depends on extracellular matrix control of myeloid suppressor cell activity. Cell Rep.

[152] Ouzounova M, Lee E, Piranlioglu R, El Andaloussi A, Kolhe R, Demirci MF, Marasco D, Asm I, Chadli A, Hassan KA (2017). Monocytic and granulocytic myeloid derived suppressor cells differentially regulate spatiotemporal tumour plasticity during metastatic cascade. Nat Commun.

[153] Weber R, Fleming V, Hu X, Nagibin V, Groth C, Altevogt P, Utikal J, Umansky V (2018). Myeloid-derived suppressor cells hinder the anti-cancer activity of immune checkpoint inhibitors. Front Immunol.

[154] Yan G, Zhao H, Zhang Q, Zhou Y, Wu L, Lei J, Wang X, Zhang J, Zhang X, Zheng L (2018). A RIPK3-PGE2 circuit mediates myeloid-derived suppressor cell-potentiated colorectal carcinogenesis. Cancer Res.

[155] Taki M, Abiko K, Baba T, Hamanishi J, Yamaguchi K, Murakami R, Yamanoi K, Horikawa N, Hosoe Y, Nakamura E (2018). Snail promotes ovarian cancer progression by recruiting myeloid-derived suppressor cells via CXCR2 ligand upregulation. Nat Commun.

[156] Li ZL, Ye SB, OuYang LY, Zhang H, Chen YS, He J, Chen QY, Qian CN, Zhang XS, Cui J (2015). COX-2 promotes metastasis in nasopharyngeal carcinoma by mediating interactions between cancer cells and myeloid-derived suppressor cells. Oncoimmunology.

[157] Wu Q, Zhang W, Wang Y, Min Q, Zhang H, Dong D, Zhan Q (2021). MAGE-C3 promotes cancer metastasis by inducing epithelial–mesenchymal transition and immunosuppression in esophageal squamous cell carcinoma. Cancer Commun.

[158] Santisteban M, Reiman JM, Asiedu MK, Behrens MD, Nassar A, Kalli KR, Haluska P, Ingle JN, Hartmann LC, Manjili MH (2009). Immune-induced epithelial to mesenchymal transition in vivo generates breast cancer stem cells. Cancer Res.

[159] Zhang Y, Yan W, Mathew E, Bednar F, Wan S, Collins MA, Evans RA, Welling TH, Vonderheide RH, di Magliano MP (2014). CD4+ T lymphocyte ablation prevents pancreatic carcinogenesis in mice. Cancer Immunol Res.

[160] Goebel L, Grage-Griebenow E, Gorys A, Helm O, Genrich G, Lenk L, Wesch D, Ungefroren H, Freitag-Wolf S, Sipos B (2015). CD4(+) T cells potently induce epithelial–mesenchymal-transition in premalignant and malignant pancreatic ductal epithelial cells-novel implications of CD4(+) T cells in pancreatic cancer development. Oncoimmunology.

[161] Chen Q, Yang D, Zong H, Zhu L, Wang L, Wang X, Zhu X, Song X, Wang J (2017). Growth-induced stress enhances epithelial–mesenchymal transition induced by IL-6 in clear cell renal cell carcinoma via the Akt/GSK-3beta/beta-catenin signaling pathway. Oncogenesis.

[162] Ma S, Yan J, Barr T, Zhang J, Chen Z, Wang LS, Sun JC, Chen J, Caligiuri MA, Yu J (2021). The RNA m6A reader YTHDF2 controls NK cell antitumor and antiviral immunity. J Exp Med.

[163] Peng LS, Zhang JY, Teng YS, Zhao YL, Wang TT, Mao FY, Lv YP, Cheng P, Li WH, Chen N (2017). Tumor-associated monocytes/macrophages impair NK-cell function via TGFbeta1 in human gastric cancer. Cancer Immunol Res.

[164] Zhang QF, Yin WW, Xia Y, Yi YY, He QF, Wang X, Ren H, Zhang DZ (2017). Liver-infiltrating CD11b(−)CD27(−) NK subsets account for NK-cell dysfunction in patients with hepatocellular carcinoma and are associated with tumor progression. Cell Mol Immunol.

[165] Makowska A, Franzen S, Braunschweig T, Denecke B, Shen L, Baloche V, Busson P, Kontny U (2019). Interferon beta increases NK cell cytotoxicity against tumor cells in patients with nasopharyngeal carcinoma via tumor necrosis factor apoptosis-inducing ligand. Cancer Immunol Immunother.

[166] Stojanovic A, Fiegler N, Brunner-Weinzierl M, Cerwenka A (2014). CTLA-4 is expressed by activated mouse NK cells and inhibits NK Cell IFN-gamma production in response to mature dendritic cells. J Immunol.

[167] Thacker G, Henry S, Nandi A, Debnath R, Singh S, Nayak A, Susnik B, Boone MM, Zhang Q, Kesmodel SB (2023). Immature natural killer cells promote progression of triple-negative breast cancer. Sci Transl Med.

[168] Hagerling C, Gonzalez H, Salari K, Wang CY, Lin C, Robles I, van Gogh M, Dejmek A, Jirstrom K, Werb Z (2019). Immune effector monocyte-neutrophil cooperation induced by the primary tumor prevents metastatic progression of breast cancer. Proc Natl Acad Sci USA.

[169] Andzinski L, Kasnitz N, Stahnke S, Wu CF, Gereke M, von Kockritz-Blickwede M, Schilling B, Brandau S, Weiss S, Jablonska J (2016). Type I IFNs induce anti-tumor polarization of tumor associated neutrophils in mice and human. Int J Cancer.

[170] Singhal S, Bhojnagarwala PS, O'Brien S, Moon EK, Garfall AL, Rao AS, Quatromoni JG, Stephen TL, Litzky L, Deshpande C (2016). Origin and role of a subset of tumor-associated neutrophils with antigen-presenting cell features in early-stage human lung cancer. Cancer Cell.

[171] Finisguerra V, Di Conza G, Di Matteo M, Serneels J, Costa S, Thompson AA, Wauters E, Walmsley S, Prenen H, Granot Z (2015). MET is required for the recruitment of anti-tumoural neutrophils. Nature.

[172] Cui C, Chakraborty K, Tang XA, Zhou G, Schoenfelt KQ, Becker KM, Hoffman A, Chang YF, Blank A, Reardon CA (2021). Neutrophil elastase selectively kills cancer cells and attenuates tumorigenesis. Cell.

[173] Butin-Israeli V, Bui TM, Wiesolek HL, Mascarenhas L, Lee JJ, Mehl LC, Knutson KR, Adam SA, Goldman RD, Beyder A (2019). Neutrophil-induced genomic instability impedes resolution of inflammation and wound healing. J Clin Invest.

[174] Veglia F, Tyurin VA, Blasi M, De Leo A, Kossenkov AV, Donthireddy L, To TKJ, Schug Z, Basu S, Wang F (2019). Fatty acid transport protein 2 reprograms neutrophils in cancer. Nature.

[175] Fridlender ZG, Sun J, Kim S, Kapoor V, Cheng G, Ling L, Worthen GS, Albelda SM (2009). Polarization of tumor-associated neutrophil phenotype by TGF-beta: “N1” versus “N2” TAN. Cancer Cell.

[176] Maas RR, Soukup K, Fournier N, Massara M, Galland S, Kornete M, Wischnewski V, Lourenco J, Croci D, Alvarez-Prado AF (2023). The local microenvironment drives activation of neutrophils in human brain tumors. Cell.

[177] Khalid F, Takagi K, Sato A, Yamaguchi M, Guestini F, Miki Y, Miyashita M, Hirakawa H, Ohi Y, Rai Y (2023). Interleukin (IL)-17A in triple-negative breast cancer: a potent prognostic factor associated with intratumoral neutrophil infiltration. Breast Cancer.

[178] Butler JM, Kobayashi H, Rafii S (2010). Instructive role of the vascular niche in promoting tumour growth and tissue repair by angiocrine factors. Nat Rev Cancer.

[179] Potente M, Makinen T (2017). Vascular heterogeneity and specialization in development and disease. Nat Rev Mol Cell Biol.

[180] Matsumoto K, Yoshitomi H, Rossant J, Zaret KS (2001). Liver organogenesis promoted by endothelial cells prior to vascular function. Science.

[181] Lammert E, Cleaver O, Melton D (2001). Induction of pancreatic differentiation by signals from blood vessels. Science.

[182] Zhang W, Xu J, Fang H, Tang L, Chen W, Sun Q, Zhang Q, Yang F, Sun Z, Cao L (2018). Endothelial cells promote triple-negative breast cancer cell metastasis via PAI-1 and CCL5 signaling. FASEB J.

[183] Ou J, Guan D, Yang Y (2019). Non-contact co-culture with human vascular endothelial cells promotes epithelial-to-mesenchymal transition of cervical cancer SiHa cells by activating the NOTCH1/LOX/SNAIL pathway. Cell Mol Biol Lett.

[184] Cassier PA, Navaridas R, Bellina M, Rama N, Ducarouge B, Hernandez-Vargas H, Delord JP, Lengrand J, Paradisi A, Fattet L (2023). Netrin-1 blockade inhibits tumour growth and EMT features in endometrial cancer. Nature.

[185] Lengrand J, Pastushenko I, Vanuytven S, Song Y, Venet D, Sarate RM, Bellina M, Moers V, Boinet A, Sifrim A (2023). Pharmacological targeting of netrin-1 inhibits EMT in cancer. Nature.

[186] Prager GW, Taieb J, Fakih M, Ciardiello F, Van Cutsem E, Elez E, Cruz FM, Wyrwicz L, Stroyakovskiy D, Papai Z (2023). Trifluridine–tipiracil and bevacizumab in refractory metastatic colorectal cancer. N Engl J Med.

[187] Seto T, Nosaki K, Shimokawa M, Toyozawa R, Sugawara S, Hayashi H, Murakami H, Kato T, Niho S, Saka H (2022). Phase II study of atezolizumab with bevacizumab for non-squamous non-small cell lung cancer with high PD-L1 expression (@Be Study). J Immunother Cancer.

[188] Jimeno A, Gordon M, Chugh R, Messersmith W, Mendelson D, Dupont J, Stagg R, Kapoun AM, Xu L, Uttamsingh S (2017). A first-in-human phase I study of the anticancer stem cell agent ipafricept (OMP-54F28), a decoy receptor for Wnt ligands, in patients with advanced solid tumors. Clin Cancer Res.

[189] Liang B, Wang H, Qiao Y, Wang X, Qian M, Song X, Zhou Y, Zhang Y, Shang R, Che L (2023). Differential requirement of Hippo cascade during CTNNB1 or AXIN1 mutation-driven hepatocarcinogenesis. Hepatology.

[190] Nandana S, Tripathi M, Duan P, Chu CY, Mishra R, Liu C, Jin R, Yamashita H, Zayzafoon M, Bhowmick NA (2017). Bone metastasis of prostate cancer can be therapeutically targeted at the TBX2-WNT signaling axis. Cancer Res.

[191] Meyerhardt JA, Shi Q, Fuchs CS, Meyer J, Niedzwiecki D, Zemla T, Kumthekar P, Guthrie KA, Couture F, Kuebler P (2021). Effect of celecoxib vs placebo added to standard adjuvant therapy on disease-free survival among patients with stage III colon cancer: the CALGB/SWOG 80702 (alliance) randomized clinical trial. JAMA.

[192] Kerr DJ, Dunn JA, Langman MJ, Smith JL, Midgley RS, Stanley A, Stokes JC, Julier P, Iveson C, Duvvuri R (2007). Rofecoxib and cardiovascular adverse events in adjuvant treatment of colorectal cancer. N Engl J Med.

[193] Fenwick SW, Toogood GJ, Lodge JP, Hull MA (2003). The effect of the selective cyclooxygenase-2 inhibitor rofecoxib on human colorectal cancer liver metastases. Gastroenterology.

[194] Cheng Y, Mo F, Li Q, Han X, Shi H, Chen S, Wei Y, Wei X (2021). Targeting CXCR2 inhibits the progression of lung cancer and promotes therapeutic effect of cisplatin. Mol Cancer.

[195] Chen H, Pan Y, Zhou Q, Liang C, Wong CC, Zhou Y, Huang D, Liu W, Zhai J, Gou H (2022). METTL3 inhibits antitumor immunity by targeting m(6)A-BHLHE41-CXCL1/CXCR2 axis to promote colorectal cancer. Gastroenterology.

[196] Cambien B, Karimdjee BF, Richard-Fiardo P, Bziouech H, Barthel R, Millet MA, Martini V, Birnbaum D, Scoazec JY, Abello J (2009). Organ-specific inhibition of metastatic colon carcinoma by CXCR3 antagonism. Br J Cancer.

[197] Walser TC, Rifat S, Ma X, Kundu N, Ward C, Goloubeva O, Johnson MG, Medina JC, Collins TL, Fulton AM (2006). Antagonism of CXCR3 inhibits lung metastasis in a murine model of metastatic breast cancer. Cancer Res.

[198] Xu B, Yan M, Ma F, Hu X, Feng J, Ouyang Q, Tong Z, Li H, Zhang Q, Sun T (2021). Pyrotinib plus capecitabine versus lapatinib plus capecitabine for the treatment of HER2-positive metastatic breast cancer (PHOEBE): a multicentre, open-label, randomised, controlled, phase 3 trial. Lancet Oncol.

[199] Saura C, Oliveira M, Feng YH, Dai MS, Chen SW, Hurvitz SA, Kim SB, Moy B, Delaloge S, Gradishar W (2020). Neratinib plus capecitabine versus lapatinib plus capecitabine in HER2-positive metastatic breast cancer previously treated with >/= 2 HER2-directed regimens: phase III NALA trial. J Clin Oncol.

[200] Watanabe J, Muro K, Shitara K, Yamazaki K, Shiozawa M, Ohori H, Takashima A, Yokota M, Makiyama A, Akazawa N (2023). Panitumumab vs bevacizumab added to standard first-line chemotherapy and overall survival among patients with RAS wild-type, left-sided metastatic colorectal cancer: a randomized clinical trial. JAMA.

[201] Sartore-Bianchi A, Pietrantonio F, Lonardi S, Mussolin B, Rua F, Crisafulli G, Bartolini A, Fenocchio E, Amatu A, Manca P (2022). Circulating tumor DNA to guide rechallenge with panitumumab in metastatic colorectal cancer: the phase 2 CHRONOS trial. Nat Med.

[202] Noronha V, Patil VM, Joshi A, Menon N, Chougule A, Mahajan A, Janu A, Purandare N, Kumar R, More S (2020). Gefitinib versus gefitinib plus pemetrexed and carboplatin chemotherapy in EGFR-mutated lung cancer. J Clin Oncol.

[203] Miyauchi E, Morita S, Nakamura A, Hosomi Y, Watanabe K, Ikeda S, Seike M, Fujita Y, Minato K, Ko R (2022). Updated analysis of NEJ009: gefitinib-alone versus gefitinib plus chemotherapy for non-small-cell lung cancer with mutated EGFR. J Clin Oncol.

[204] Charan M, Dravid P, Cam M, Audino A, Gross AC, Arnold MA, Roberts RD, Cripe TP, Pertsemlidis A, Houghton PJ (2020). GD2-directed CAR-T cells in combination with HGF-targeted neutralizing antibody (AMG102) prevent primary tumor growth and metastasis in Ewing sarcoma. Int J Cancer.

[205] Agarwal N, McGregor B, Maughan BL, Dorff TB, Kelly W, Fang B, McKay RR, Singh P, Pagliaro L, Dreicer R (2022). Cabozantinib in combination with atezolizumab in patients with metastatic castration-resistant prostate cancer: results from an expansion cohort of a multicentre, open-label, phase 1b trial (COSMIC-021). Lancet Oncol.

[206] Esteban-Fabro R, Willoughby CE, Pique-Gili M, Montironi C, Abril-Fornaguera J, Peix J, Torrens L, Mesropian A, Balaseviciute U, Miro-Mur F (2022). Cabozantinib enhances anti-PD1 activity and elicits a neutrophil-based immune response in hepatocellular carcinoma. Clin Cancer Res.

[207] Sterner RM, Sakemura R, Cox MJ, Yang N, Khadka RH, Forsman CL, Hansen MJ, Jin F, Ayasoufi K, Hefazi M (2019). GM-CSF inhibition reduces cytokine release syndrome and neuroinflammation but enhances CAR-T cell function in xenografts. Blood.

[208] Kantoff PW, Higano CS, Shore ND, Berger ER, Small EJ, Penson DF, Redfern CH, Ferrari AC, Dreicer R, Sims RB (2010). Sipuleucel-T immunotherapy for castration-resistant prostate cancer. N Engl J Med.

[209] Shalhout SZ, Miller DM, Emerick KS, Kaufman HL (2023). Therapy with oncolytic viruses: progress and challenges. Nat Rev Clin Oncol.

[210] Hong C, Schubert M, Tijhuis AE, Requesens M, Roorda M, van den Brink A, Ruiz LA, Bakker PL, van der Sluis T, Pieters W (2022). cGAS-STING drives the IL-6-dependent survival of chromosomally instable cancers. Nature.

[211] Song L, Smith MA, Doshi P, Sasser K, Fulp W, Altiok S, Haura EB (2014). Antitumor efficacy of the anti-interleukin-6 (IL-6) antibody siltuximab in mouse xenograft models of lung cancer. J Thorac Oncol.

[212] Dijkgraaf EM, Santegoets SJ, Reyners AK, Goedemans R, Wouters MC, Kenter GG, van Erkel AR, van Poelgeest MI, Nijman HW, van der Hoeven JJ (2015). A phase I trial combining carboplatin/doxorubicin with tocilizumab, an anti-IL-6R monoclonal antibody, and interferon-alpha2b in patients with recurrent epithelial ovarian cancer. Ann Oncol.

[213] Karkera J, Steiner H, Li W, Skradski V, Moser PL, Riethdorf S, Reddy M, Puchalski T, Safer K, Prabhakar U (2011). The anti-interleukin-6 antibody siltuximab down-regulates genes implicated in tumorigenesis in prostate cancer patients from a phase I study. Prostate.

[214] Sullivan KM, Jiang X, Guha P, Lausted C, Carter JA, Hsu C, Labadie KP, Kohli K, Kenerson HL, Daniel SK (2023). Blockade of interleukin 10 potentiates antitumour immune function in human colorectal cancer liver metastases. Gut.

[215] Lamichhane P, Karyampudi L, Shreeder B, Krempski J, Bahr D, Daum J, Kalli KR, Goode EL, Block MS, Cannon MJ (2017). IL10 release upon PD-1 blockade sustains immunosuppression in ovarian cancer. Cancer Res.

[216] Kawano Y, Zavidij O, Park J, Moschetta M, Kokubun K, Mouhieddine TH, Manier S, Mishima Y, Murakami N, Bustoros M (2018). Blocking IFNAR1 inhibits multiple myeloma-driven Treg expansion and immunosuppression. J Clin Invest.

[217] Sinha P, Clements VK, Fulton AM, Ostrand-Rosenberg S (2007). Prostaglandin E2 promotes tumor progression by inducing myeloid-derived suppressor cells. Cancer Res.

[218] Jeong JW, Park C, Cha HJ, Hong SH, Park SH, Kim GY, Kim WJ, Kim CH, Song KS, Choi YH (2018). Cordycepin inhibits lipopolysaccharide-induced cell migration and invasion in human colorectal carcinoma HCT-116 cells through down-regulation of prostaglandin E2 receptor EP4. BMB Rep.

[219] Jiang L, Zhao XH, Mao YL, Wang JF, Zheng HJ, You QS (2019). Long non-coding RNA RP11–468E2.5 curtails colorectal cancer cell proliferation and stimulates apoptosis via the JAK/STAT signaling pathway by targeting STAT5 and STAT6. J Exp Clin Cancer Res.

[220] Zhou X, Yan T, Huang C, Xu Z, Wang L, Jiang E, Wang H, Chen Y, Liu K, Shao Z (2018). Melanoma cell-secreted exosomal miR-155-5p induce proangiogenic switch of cancer-associated fibroblasts via SOCS1/JAK2/STAT3 signaling pathway. J Exp Clin Cancer Res.

[221] Zhang L, Nesvick CL, Day CA, Choi J, Lu VM, Peterson T, Power EA, Anderson JB, Hamdan FH, Decker PA (2022). STAT3 is a biologically relevant therapeutic target in H3K27M-mutant diffuse midline glioma. Neuro Oncol.

[222] Sun S, Wu Y, Guo W, Yu F, Kong L, Ren Y, Wang Y, Yao X, Jing C, Zhang C (2018). STAT3/HOTAIR signaling axis regulates HNSCC growth in an EZH2-dependent manner. Clin Cancer Res.

[223] Xia X, Zhang Z, Zhu C, Ni B, Wang S, Yang S, Yu F, Zhao E, Li Q, Zhao G (2022). Neutrophil extracellular traps promote metastasis in gastric cancer patients with postoperative abdominal infectious complications. Nat Commun.

[224] Rodon J, Carducci MA, Sepulveda-Sanchez JM, Azaro A, Calvo E, Seoane J, Brana I, Sicart E, Gueorguieva I, Cleverly AL (2015). First-in-human dose study of the novel transforming growth factor-beta receptor I kinase inhibitor LY2157299 monohydrate in patients with advanced cancer and glioma. Clin Cancer Res.

[225] David CJ, Huang YH, Chen M, Su J, Zou Y, Bardeesy N, Iacobuzio-Donahue CA, Massague J (2016). TGF-beta tumor suppression through a lethal EMT. Cell.

[226] Melisi D, Oh DY, Hollebecque A, Calvo E, Varghese A, Borazanci E, Macarulla T, Merz V, Zecchetto C, Zhao Y (2021). Safety and activity of the TGFbeta receptor I kinase inhibitor galunisertib plus the anti-PD-L1 antibody durvalumab in metastatic pancreatic cancer. J Immunother Cancer.

[227] Smyth EC, Nilsson M, Grabsch HI, van Grieken NC, Lordick F (2020). Gastric cancer. Lancet.

[228] Reckamp KL, Redman MW, Dragnev KH, Minichiello K, Villaruz LC, Faller B, Al Baghdadi T, Hines S, Everhart L, Highleyman L (2022). Phase II randomized study of ramucirumab and pembrolizumab versus standard of care in advanced non-small-cell lung cancer previously treated with immunotherapy-lung-MAP S1800A. J Clin Oncol.

[229] Xie H, Lafky JM, Morlan BW, Stella PJ, Dakhil SR, Gross GG, Loui WS, Hubbard JM, Alberts SR, Grothey A (2020). Dual VEGF inhibition with sorafenib and bevacizumab as salvage therapy in metastatic colorectal cancer: results of the phase II North Central Cancer Treatment Group study N054C (Alliance). Ther Adv Med Oncol.

[230] Gray RT, Cantwell MM, Coleman HG, Loughrey MB, Bankhead P, McQuaid S, O'Neill RF, Arthur K, Bingham V, McGready C (2017). Evaluation of PTGS2 expression, PIK3CA mutation, aspirin use and colon cancer survival in a population-based cohort study. Clin Transl Gastroenterol.

[231] Dotan E, Cardin DB, Lenz HJ, Messersmith W, O'Neil B, Cohen SJ, Denlinger CS, Shahda S, Astsaturov I, Kapoun AM (2020). Phase Ib study of Wnt inhibitor ipafricept with gemcitabine and nab-paclitaxel in patients with previously untreated stage IV pancreatic cancer. Clin Cancer Res.

[232] Pistore C, Giannoni E, Colangelo T, Rizzo F, Magnani E, Muccillo L, Giurato G, Mancini M, Rizzo S, Riccardi M (2017). DNA methylation variations are required for epithelial-to-mesenchymal transition induced by cancer-associated fibroblasts in prostate cancer cells. Oncogene.

[233] Yu Y, Xiao CH, Tan LD, Wang QS, Li XQ, Feng YM (2014). Cancer-associated fibroblasts induce epithelial–mesenchymal transition of breast cancer cells through paracrine TGF-beta signalling. Br J Cancer.

[234] Zhao L, Ji G, Le X, Luo Z, Wang C, Feng M, Xu L, Zhang Y, Lau WB, Lau B (2017). An integrated analysis identifies STAT4 as a key regulator of ovarian cancer metastasis. Oncogene.

[235] Ben Baruch B, Mantsur E, Franco-Barraza J, Blacher E, Cukierman E, Stein R (2020). CD38 in cancer-associated fibroblasts promotes pro-tumoral activity. Lab Invest.

[236] Liu M, Kuo F, Capistrano KJ, Kang D, Nixon BG, Shi W, Chou C, Do MH, Stamatiades EG, Gao S (2020). TGF-beta suppresses type 2 immunity to cancer. Nature.

[237] Seebauer CT, Brunner S, Glockzin G, Piso P, Ruemmele P, Schlitt HJ, Geissler EK, Fichtner-Feigl S, Kesselring R (2016). Peritoneal carcinomatosis of colorectal cancer is characterized by structural and functional reorganization of the tumor microenvironment inducing senescence and proliferation arrest in cancer cells. Oncoimmunology.

